# Human impact on the recent population history of the elusive European wildcat inferred from whole genome data

**DOI:** 10.1186/s12864-022-08930-w

**Published:** 2022-10-18

**Authors:** María Esther Nieto-Blázquez, Dennis Schreiber, Sarah A. Mueller, Katrin Koch, Carsten Nowak, Markus Pfenninger

**Affiliations:** 1 Molecular Ecology Group, Senckenberg Biodiversity and Climate Research Centre (SBiK-F), 60325 Frankfurt am Main, Germany; 2 Division of Evolutionary Biology, Faculty of Biology, Ludwig Maximilian University of Munich, Planegg-Martinsried 82152, Munich, Germany; 3 Centre for Wildlife Genetics, Senckenberg Research Institute and Natural History Museum Frankfurt, 63571 Gelnhausen, Germany; 4 European Wildcat Monitoring, Bund Für Umwelt Und Naturschutz, Rheinland-Pfalz, 55118 Mainz, Germany; 5 LOEWE Centre for Translational Biodiversity Genomics (LOEWE-TBG), 60325 Frankfurt am Main, Germany; 6 Institute for Molecular and Organismic Evolution, Johannes Gutenberg University, 55128 Mainz, Germany

**Keywords:** *Felis silvestris*, Whole-genome sequencing, Anthropogenic disturbance, Population divergence, Adaptive selection, Introgression, Conservation genomics

## Abstract

**Background:**

The extent and impact of evolutionary change occurring in natural populations in response to rapid anthropogenic impact is still poorly understood on the genome-wide level. Here, we explore the genetic structure, demographic history, population differentiation, and domestic introgression based on whole genome data of the endangered European wildcat in Germany, to assess potential genomic consequences of the species’ recent spread across human-dominated cultural landscapes.

**Results:**

Reconstruction of demographic history and introgression rates based on 47 wildcat and 37 domestic cat genomes suggested late introgression between wild and domestic cat, coinciding with the introduction of domestic cat during the Roman period, but overall relatively low rates of hybridization and introgression from domestic cats. Main population divergence found between an eastern and central German wildcat clade was found to be of rather recent origin (200 y), and thus the likely consequence of anthropogenic persecution and resulting isolation in population refugia. We found similar effective population sizes and no substantial inbreeding across populations. Interestingly, highly differentiated genes between wild cat populations involved in the tryptophan-kynurenine-serotonin pathway were revealed, which plays a role in behavioral processes such as stress susceptibility and tolerance, suggesting that differential selection acted in the populations.

**Conclusions:**

We found strong evidence for substantial recent anthropogenic impact on the genetic structure of European wildcats, including recent persecution-driven population divergence, as well as potential adaptation to human-dominate environments. In contrast, the relatively low levels of domestic introgression and inbreeding found in this study indicate a substantial level of “resistance” of this elusive species towards major anthropogenic impacts, such as the omnipresence of domestic cats as well as substantial habitat fragmentation. While those findings have strong implications for ongoing conservation strategies, we demand closer inspection of selective pressures acting on this and other wildlife species in anthropogenic environments.

**Supplementary Information:**

The online version contains supplementary material available at 10.1186/s12864-022-08930-w.

## Background

The anthropogenic alteration of ecosystems has been acknowledged as the single greatest threat to global biodiversity [1, 2], leaving a footprint at an unprecedented rate [3]. Human impact is also a well-recognized evolutionary force [4, 5], and plenty of evidence is now available on how human disturbances alter selective pressures on populations [6–8], which results in greater and more rapid phenotypic selection than would happen in natural populations [6, 9, 10]. Threats to wildlife populations often act in a synergistic way, and genetic factors are fundamental to understand anthropogenic impact on wildlife populations and assist in the efforts to mitigate the challenges wildlife might confront [11]. As seen for several wildlife species (e.g. soay sheep, gray wolves or wildcat), introgression with domestic congeners is an indirect form of human-mediated evolution, which can pose a major conservation threat for wildlife, and indeed led to the extinction of many plants and animals [12, 13]. Despite of this human impact, many species benefit from human-modified environments, such as synanthropic species (e.g. masked palm civet, raccoon dog or red foxes [14], which show phenotypical plasticity and increased population densities [15]. In addition, several historically persecuted wildlife species of large (e.g., brown bear, gray wolf, Eurasian lynx) and medium-sized (e.g., Eurasian otter, European wildcat) carnivores currently increase in range and abundance throughout several regions, including a successful ongoing spread within the densely populated, anthropogenically transformed landscapes of Western and Central Europe.

The European wildcats (*Felis silvestris,* hereafter *FS*), for instance, suffered considerable range declines and population reductions in Central Europe, due to anthropogenic persecution as well as habitat loss and fragmentation during the 19^th^ and early twentieth century [16, 17]. Recent studies show that despite the fragmented and mosaic-like German landscape, the species has recently reemerged in various regions [18, 19], and range expansion is still ongoing and even comprises areas with no historic presence data of *F. silvestris* [20].

Previous studies using traditional marker systems (i.e., mitochondrial control region sequences and microsatellites; [21–23]) show that wildcats in Germany are currently clustered into two main groups, a western and central one. However, little is known about the historical processes that have led to the current allopatric distribution, with no obvious landscape barrier separating both populations [18, 24]. European wildcats survived Pleistocene glaciations in three main Mediterranean refugia in southern Iberian, Italy, and Balkan peninsulas, followed by postglacial northward recolonizations [21]. This study concluded that the two populations observed in Germany are most probably due to recolonization events from different refugia. evidence, however, suggests a recent origin of population divergence, perhaps as a consequence of massive persecution and re-expansion from different historic refugia [20].

While domestic cats (*Felis catus,* hereafter *FC*) do not directly derive from European wildcats (*FS*), successful hybridization between FS and FC has been found in various regions of their distribution. Particularly high levels of hybridization have been revealed in Scotland and Hungary [21], whereas rather low degrees of admixture were found in other European regions [21–23]. The different admixture level amongst regions can be explained by variation in population histories [21, 25], and the disparity in ecological barriers and environmental conditions [26]. However, despite of low observed recent hybridization in certain European regions, the potential effects of long-term introgression of domestic cat DNA into the genepool of *F. silvestris* is still largely unknown. Archaeozoological data suggests the presence of domestic cat in central Europe at least during the Roman Period, ca. 2,000 ya, introduced by either Celts or Romans [27], which expanded extensively during Medieval times as a critical aid against vermin and pests [28] and was included in trades for its pelt [29]. Thus, long-term impact of domestic cat presence, in particular within the Roman-occupied areas in Germany, might have resulted in the accumulation of domestic cat ancestry within wildcat genomes, and potentially even contribute to the observed population divergence.

Here we apply a population genomic approach based on whole genome data from wildcat samples across Germany to reconstruct recent population history and assess anthropogenic impacts on the genetic integrity of the species. With the advent of high-throughput sequencing methods, conservation genomics has the potential to assist with identifying historical *versus* contemporary scenarios of population differentiation for non-model organisms [30] and also patterns of hybridization and introgression [31]. The viability of local populations of conservation concern that are impacted by climatic change and anthropogenic activities and their ability to adapt to such challenges can be predicted by identifying regions of the genomes under selection [32]. In this study we focus on i) the temporal pattern of differentiation among wildcat populations by historic (e.g. introduction of domestic cat by romans, spread of domestic cat during medieval age or Pleistocene glacial refugia) rather than contemporary events (e.g. anthropogenic pressures), ii) the selective pressures that may be associated to human influence, iii) the current level of genome-wide genetic diversity and inbreeding across the species range in Germany, which may reflect historic and recent anthropogenic impact, and iv) the proportion of domestic cat introgression and the potential role of introgression in the divergence of the two German wildcat groups. Answering those questions is of pivotal importance to understand how populations adapt and survive in human landscapes, which may have considerable implications for conservation strategies in humans-impacted areas.

## Results

### Whole-genome sequencing and population structure

We generated whole-genome sequencing data for 47 wildcat and 16 domestic cat individuals of a mean coverage of 18-25X. A total of 38,090,943 SNPs (single nucleotide polymorphisms) were found in the full data set containing wildcat and domestic cat, and 28,618,073 SNPs after passing filtering steps.

**Fig. 1:**
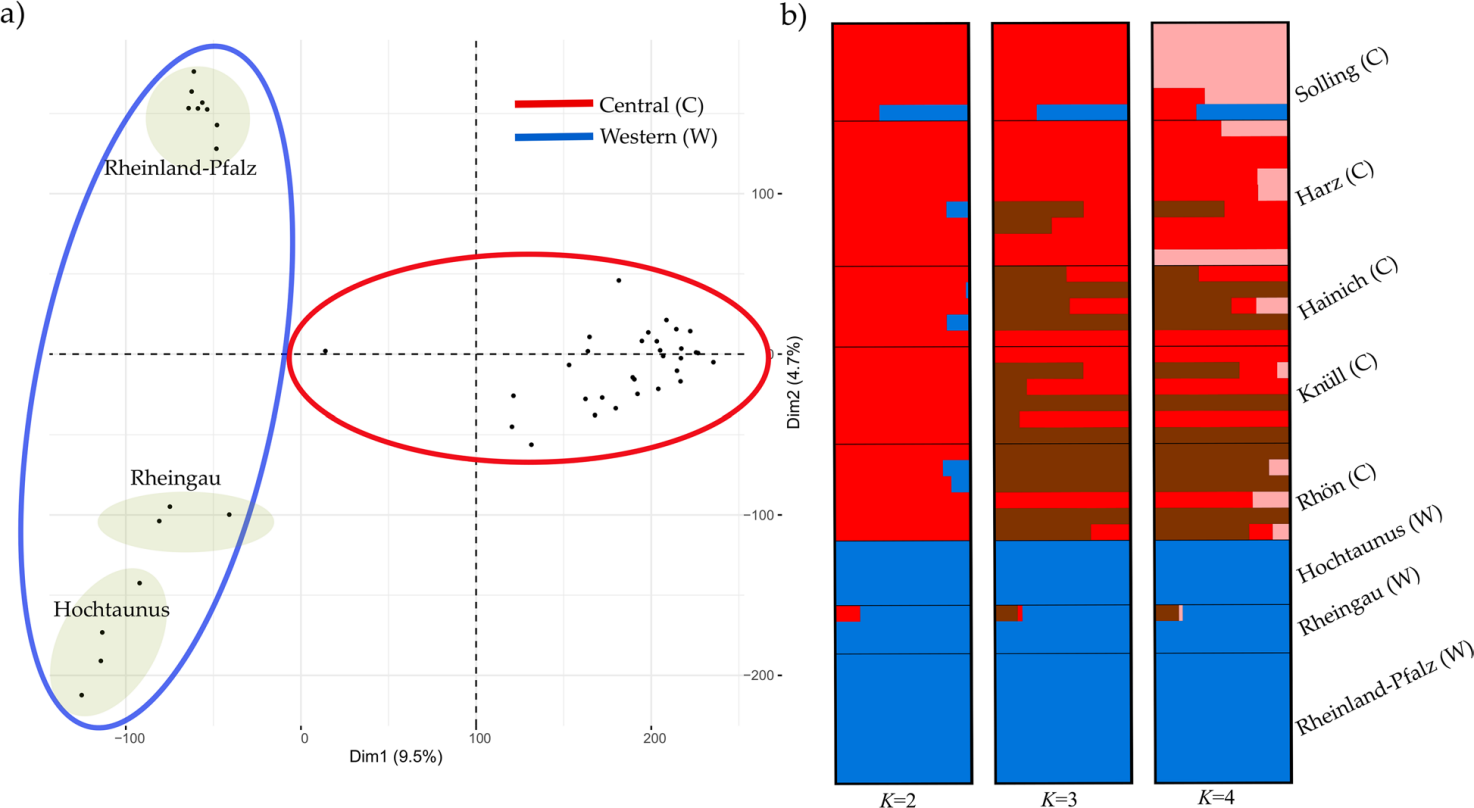
**a** Principal component analysis (PCA) based on the wildcats dataset only; **b** ADMIXTURE population structure plots for wildcats only for *K* = 2, *K* = 3 and *K* = 4. Each bar (x-axis) represents a single individual. Coloring (y-axis) corresponds to the estimated posterior probabilities proportions assignments of each individual to each *K* cluster

ADMIXTURE showed a clear structure for the dataset including domestic cat and wildcat (*K* = 2, Figure S1, Supporting Information) with a log-likelihood value = -71,316,531.6 and lowest cross validation (CV)-error = 0.312. When only wildcat were analyzed, both ADMIXTURE and principal component analysis (PCA) methods found statistical support for two clusters (*K* = 2, Fig. 1a) with a log-likelihood value = -17,823,710.6 and lowest CV-error = 0.432. The PCA approach clustered individuals according to the a priori location assignment (Fig. 1b). PC1 explained 9.5% of the total variance and split Western and Central wildcats, whereas PC2, explaining 4.7% of the total variance, showed a split between Rheinland-Pfalz individuals and, the Rheingau and Hochtaunus region, which are separated by the Rhine river. Our results do not show an apparent clustering within the Western cluster group. Individual “FB021A” from Solling appeared as an intermediate individual and did cluster neither with Western nor Central wildcats.

### Historical demography and scenarios of divergence

Both analyses, PSMC and MSMC2, recovered clearly different demographic histories for the two species and much lower effective population size (*N*_*e*_) for wildcats in comparison to domestic cats. The PSMC analysis showed no significantly different demographic trajectories between Central and Western wildcat individuals, indicating the existence of a single demographic population for most of the time. Results showed a steep *N*_*e*_ growth of wild cats in the Middle-Pleistocene (ca. 400 thousand years ago (kya)) reaching the peak in the Upper-Pleistocene with an average *N*_*e*_ of 70,000 individuals (ca. 110 kya), and followed by a continuous decrease since then (Figure S2a and, Figure S3 for bootstrap ranges). The MSMC2 analysis yielded extremely high *N*_*e*_ values which we do not take as face values but rather as a relative measure. However, there was indication of a steep and sudden drop after the postglacial demographic increase a few hundred years ago. Results also showed the different postglacial trajectories of the two wildcat populations and the relative difference between timing and drop in *N*_*e*_ (Figure S2b).

**Fig. 2:**
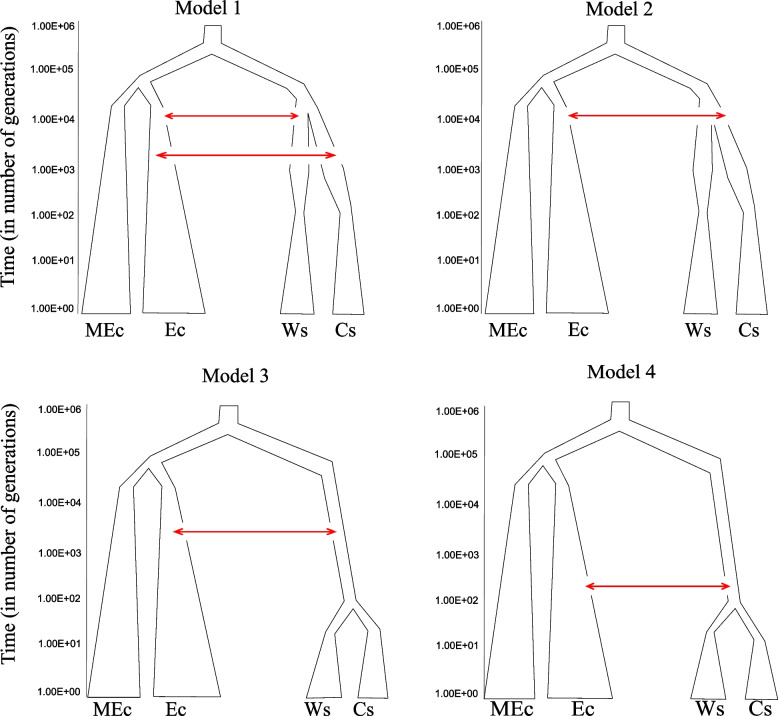
Schematic diagram showing the four historical demographic models compared in fastsimcoal2 v.2.6.0.3. MEc = Middle Eastern domestic cat; Ec = European domestic cat; Ws = Western Germany wildcat; and Cs = Central Germany wildcat. Red arrows indicate introgression

The Approximate Bayesian Computation (ABC) demographic analysis recovered a proportion of accepted simulations for all demographic models tested. Demographic model 3, which hypothesized a late split of western and central wildcat populations and early introgression, received the lowest support of all. The best-supported demographic model was model 4 (Fig. 2), with 95% of accepted simulations of (Table 1).

**Table 1:** Model selection from fastsimcoal2 analyses. Bold indicates the demographic scenario that received the highest support*. pp*’s = posterior probabilities

**Demographic model**	**% of simulations accepted** (*pp*'s)
M1	0.0325
M2	0.0075
M3	0.005
**M4**	**0.955**

Model 4 suggested a late introgression event (ca. 600 generations ago) and a recent split (ca. 100 generations ago) of western and central wildcat populations (Table S1). Second best model (Model 1, 3% of accepted simulations, Table 1) suggested also an early split (ca. 3500 generations ago) of western and central wildcats and differential introgression. Together, models with a very recent split of western and central wildcat populations received > 95% support. The best tested model (Model 4) provides a good fit to the data (Figure S4, Supporting Information).

### Inbreeding: runs of homozygosity

The proportion of the genome that was contained in runs of homozygosity (F_ROH_) ranged between 0.004 and 0.2 (Figure S5 in Supp. Information). There were no significant differences in ROH measurements between the West and Central populations, nor significant regional structuring (*P* = 0.39, 0.72). Analysis of long ROH (> 2 Mb and > 5 Mb), which can detect recent rather than distant inbreeding, identified 1,771 ROH segments in 45 individuals and 57 segments in 30 individuals, respectively. Thus, while at least some degree of recent inbreeding was detected in 63% of individuals, it was not tied to population structuring within and between populations.

### Selection analyses and functional enrichment analyses

**Fig. 3:**
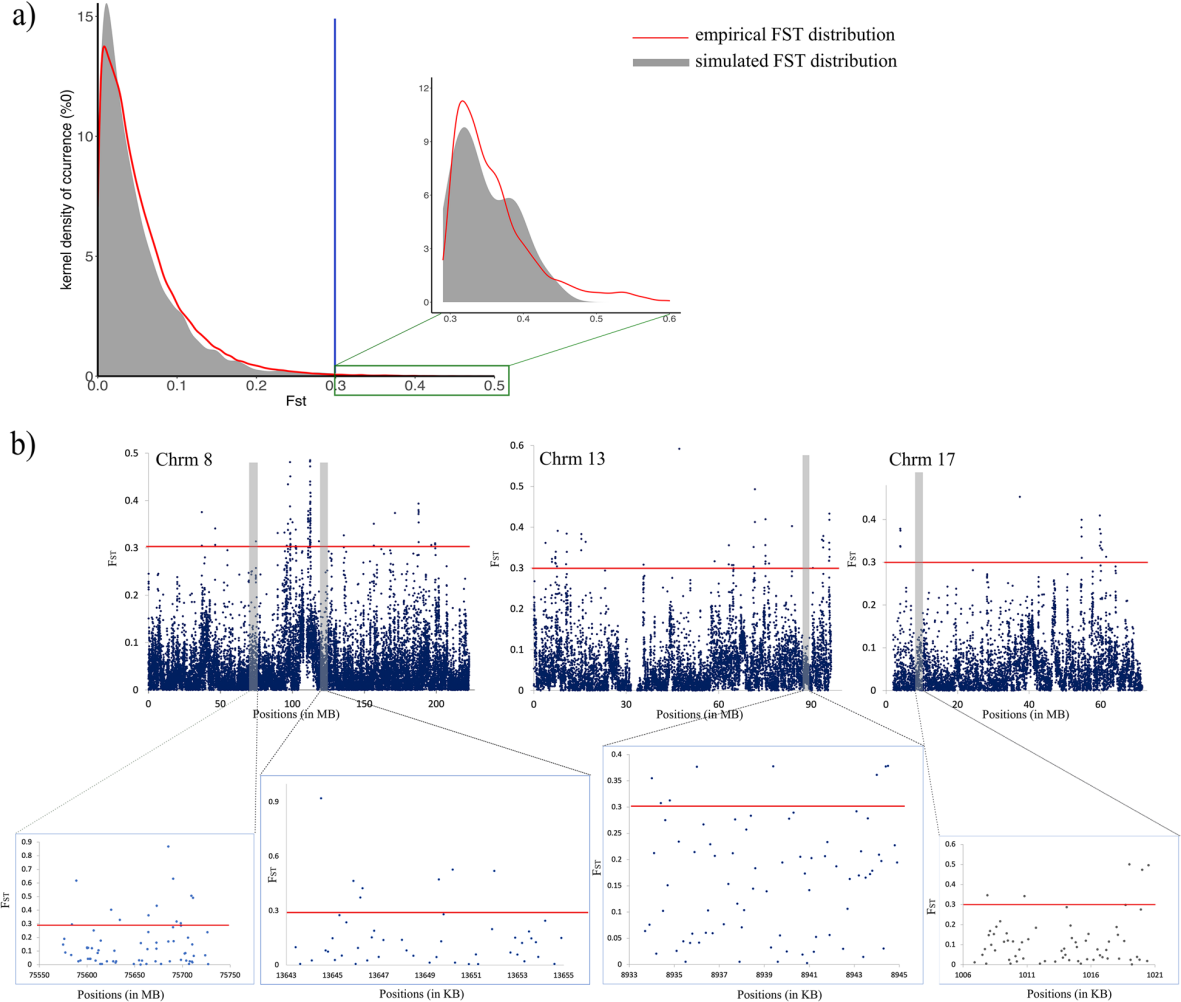
**a** Density plot representing the distribution of empirical (red) and simulated (grey) *F*_ST_ distributions; **b** *F*_ST_ above 99% threshold of the four genes across three chromosomes obtained from enrichment analyses

Empirical and simulated *F*_ST_ distributions between Western and Central wildcats were similar, with simulated distribution having a higher kernel density of occurrences (Fig. 3a). We found 64 genes with *F*_ST_ values above the 99% quantile of the simulated *F*_ST_. These genes were therefore considered as potential candidate genes under selection. Associated with the 64 candidate genes, 16 gene ontology (GO) terms were significantly enriched for biological processes (BP -Table S2, Supporting Information). After applying the FDR correction only two GO terms remained statistically significant.

Four genes associated with two significantly overrepresented top GO terms (Fig. 3b and Table S2) were associated with kynurenine metabolic and tryptophan catabolic processes. In particular, these genes were involved in the coding of cysteine-S-conjugate beta-lyase 2, kynureninase, kyrenina aminotransferase 1, and kynurenine 3-monooxygenase (tryptophan-kynurerine-serotonin pathway in Figure S6, Supporting Information).

### Haplotype relationships

Sequence length (in bp) and number of polymorphic sites for the four genes identified in the selection analyses were 77,142 and 34, 111,195 and 59, 35473 and 58, and 57,619 and 33, respectively. A total of 17, 18, 0, and 21 unique haplotypes were found for genes 1–4 respectively. The haplotype network diagrams for genes 1–3 show a clear grouping of wildcat vs domestic cats haplotypes, except for gene four, for which there is not that apparent structure. Almost no haplotypes were shared between wild and domestic cats. Overall, the number of haplotypes shared amongst the three groups was insignificant, while shared haplotypes between Central and Western wildcats were more frequent (Figure S7 in Supp. Information).

### Introgression between *FS* and *FC*

**Fig. 4:**
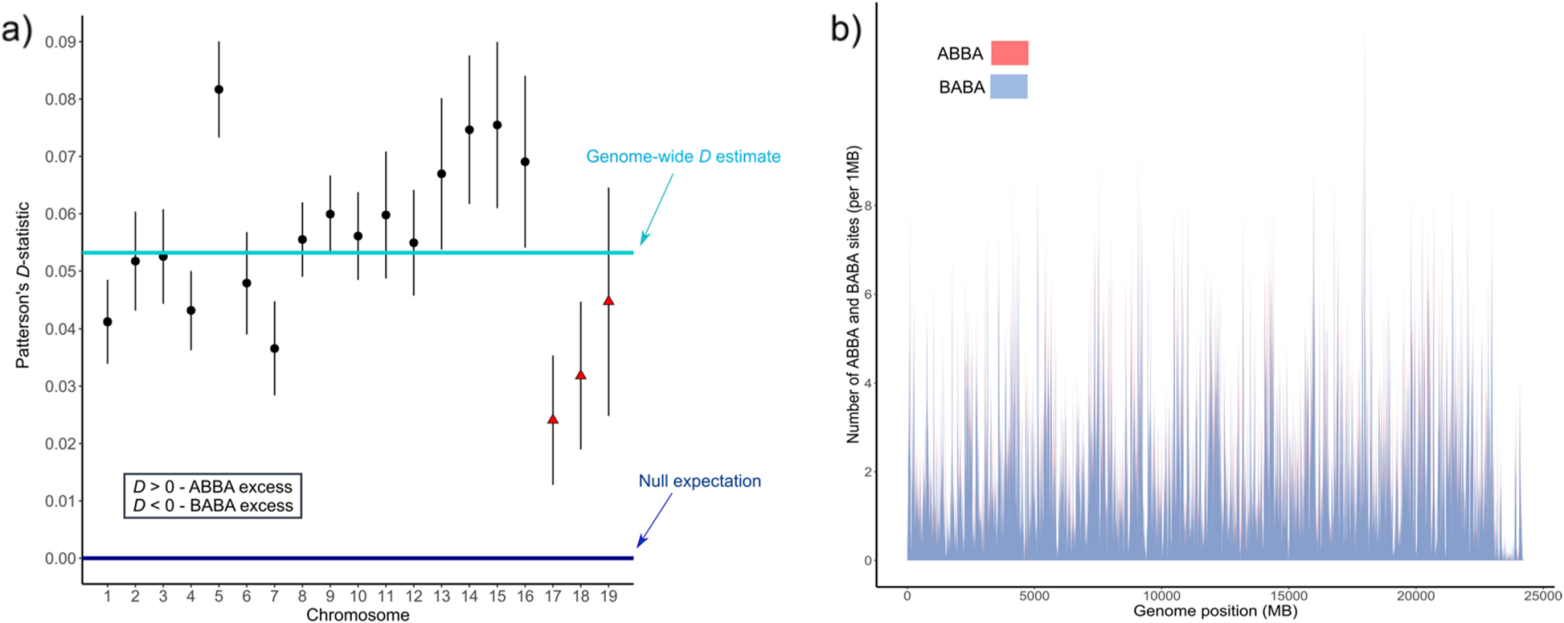
**a** Patterson’s D-statistic (± s.e.) amongst chromosomes. The dark blue line indicates no deviation from the true evolutionary history topology [(((*Central wildcat, Western wildcat*), *domestic cat*), *outgroup*)]; the light blue colored line indicates genome-wide *D*-statistic; black circles indicate significant deviation from 0 (Z-score > 3–4); and red triangles indicate non-significant deviations from 0 (Z-score < 3–4); **b** number of ABBA and BABA sites across SNPs in 10 Mb sliding windows

The genome-wide ABBA-BABA test identified a very slight excess of the ABBA pattern (Fig. 4a - D = 0.0532), indicating introgression between Western wildcat and domestic cat. The Z score (Z = 24.84) and *p*-value (*p*-value = 3.302708e-136) were significant for the *D* estimation. The proportion of the genome introgressed between Western wildcat and domestic cat was relatively low (*f* = 0.08855515; 95% confidence interval 0.0812–0.0959), however, the proportion of the genome introgressed between Central wildcat and domestic cat remains unknown. ABBA and BABA site counts across the genome were similar (Fig. 4b) and the chromosome level analysis also identified an excess of ABBA pattern (Fig. 4a - D estimation for all chromosomes > 0). However, Z score was not significant (Z < 3–4) for chromosomes 17–19 (Table S3 in Supp. Information).

## Discussion

In this study, we used WGS data for the first time to investigate the demographic history of German wildcats and the potential role of hybridization with domestic cat or other anthropogenic effects on population differentiation. Our analyses showed that the split of western and central groups of *FS* in Germany was quite recent, probably due to anthropogenic impact, rather than ancient historical events as previously believed and most likely not due to differential introgression.

### Phylogeographic structure and historical demography of German wildcats

Our results from the population structure analyses clearly showed two groups of German wildcats that corresponded to western and central locations, which are in agreement with previous studies that have used limited genetic information from microsatellites or reduced SNP panels [18, 21–23, 33]. The PCA clustering analysis further separated the western group into three subgroups, which corresponded to Rheinland-Pfalz (west of Rhine River), Rheingau and Hochtaunus locations (east of Rhine). In contrast to [18, 24], our analyses did not recover the subgroups Rheingau and Hochtaunus, as expected due to major traffic infrastructures which act as gene flow barriers. In accordance with [18], our PCA analysis showed no substantial substructure in the central cluster (Fig. 1a and Figure S1).

The model-based clustering method showed an unexpected highly homogeneous western group, with just one genetic pool recovered even at higher *K*s (Fig. 1b and Figure S1). One potential explanation might the high forest cover and low grades of habitat fragmentation in the western part of the species’ range in Germany that still led to substantial genetic exchange. However, we cannot discard the possibility that ADMIXTURE did not reveal substructure in the western cluster due to the small sample size per location. In contrast, central wildcats showed considerable differentiation when only wildcats are analyzed (Fig. 1b), which likely corresponds to geographic differentiation during the strong bottleneck period in the late 19^th^ and early twentieth century [20]. In addition, founder effects resulting from recent expansions and reintroductions, for example in the Rhön Mountains [19], might have contributed to the currently observed spatial genetic substructuring. Our study shows that wildcats in Germany do not show signs of substantial inbreeding or genetic depletion, which is likely due to the ongoing recovery after cessation of the massive persecution since the second half of the twentieth century [20]. In contrast, relatively long ROHs values were found for single individuals (Figure S5b), which suggest that inbreeding may occur occasionally in all populations.

Effective population sizes (*N*_*e*_) for western and central German wildcat have experienced subtle changes, but generally, western and central wildcats present similar *N*_*e*_. There is also no population separation recovered by the PSMC analyses for the period where this analysis yielded sufficient resolution i.e. before 10000 years ago (Figure S2a). As seen for other organisms, the N_*e*_ peak for wildcats occurred towards the end of the last interglacial period (ca. 110 kya), which was a period of the Late Pleistocene characterized by wetter and warmer environmental conditions [34–36]. This period also coincides with the upper limit of the Middle to Late Pleistocene where an increase of small-sized carnivores is observed as a consequence of the climate oscillations [37]. The *N*_*e*_ inference using MSMC2 is most likely biased by phasing errors and results should be taken cautiously. It has been shown that phasing errors in haplotype-based inference methods, such MSMC, break up identity-by-state tracts in closely related haplotypes which leads to inferring large recent effective population sizes [38].

The Approximate Bayesian computation analysis supported a relatively recent introgression between wildcats and European domestic cats, and a only very recent split of western and central wildcat populations around 100 generations ago (Model 4, Fig. 2). The late introgression suggested by the best fit model ca. 700–1,000 generations ago occurred not directly upon the time of the domestic cat introduction by the Romans, but rather during the medieval spread of cats as primary domestic mousers. Our results indicated that the divergence of western and central wildcat groups occurred rather recently. In principle, a population split can be due to loss of habitat (including land-use changes) or a demographic decline, leading to extirpation of local populations and eventually isolation of relic populations in refugia [39]. Even though overall wood cover in Europe did not change significantly since 1600, changed forestry practices might have negatively impacted wildcats demography [40]. In particular the shift from unmanaged or coppiced deciduous woods to managed coniferous high stands from the mid-nineteenth century onwards [40] probably meant a significant habitat reduction for the species [41–43]. The most likely reason for the observed split, however, is a demographic decline due to anthropogenic hunting pressure. Persecution of wild cats probably increased with the widespread introduction of effective hunting shotguns in the second half of the eighteenth century [44], augmented by societal changes in hunting privileges [45]. Both the broad availability of an effective hunting weapon for this small elusive carnivore as well as the increase in the number of hunters by permitting bourgeoise and peasant hunting likely led to a rapid decline of the species, perceived as pest and competitor for prey.

This finding of a very recent, anthropogenically induced split between western and central wildcats is in line with results based on museum material of wildcats from Germany. A recent study [20] showed that historic material collected before World War II from across Germany did not display any signals of population differentiation. This is in line with our findings and strongly suggests that the emergence of the central wildcat population is a consequence of historic persecution and a resulting severe bottleneck and subsequent re-expansion in recent decades [20]. Interestingly, previous studies based only on microsatellite markers had erroneously concluded that the divergence between western and central wildcats derive rather from more ancient, natural processes of glacial isolation, and suggested to treat both lineages as divergent clades [21, 25]. Correcting this assumption has important conservation implications for this endangered species (see below).

### Functional basis of population divergence

Some of the highly differentiated genes are involved in the tryptophan-kynurenine-serotonin pathway, in particular in the catabolism of secondary metabolites and production of nicotinamide metabolism (NAD). The fact that the differentiated genes are ultimately involved in the serotonin production might have important behavioral implications on the wildcats. Tryptophan is the metabolic precursor to serotonin, which is a neuromodulator involved in the regulation of several behavioral processes, including aggression, mood, and stress susceptibility and -resistance in both vertebrates and invertebrates [46, 47]. In fact, supplementing diet with tryptophan is widely used in domestic and livestock animals to modulate aggressiveness, hyperactivity and stress recovery [48–51]. 

It is widely accepted that human activities may negatively impact and have fitness consequences to wildlife, including stress, disease and reproductive success [52–54]. With the evidence from our study, we were not able to identify whether the production of serotonin is actually upregulated or downregulated in the wild cats. Neither could we determine with the data at hand whether one of the two German wildcat clusters might display a higher stress tolerance. Therefore, investigating different wildcat genotypes regarding stress tolerance/resistance level would shed more light into this issue. We offer two potential, mutually non-exclusive hypotheses to the observed differentiation at these genes in German wildcat populations: 1) reintroductions in one of the two populations have substantially supplemented populations with animals that have been previously raised and kept in captivity. Long-term close human contact in zoos might have led to the selection of stress resistance in these wildcats – more heritably stress resistant individuals might have better survived and reproduced in captivity, 2) an increased frequency of human impact within wildcat habitats (e.g. leisure activities like hiking, mountain biking, geochaing etc.) during the past decades might have posed selection pressures for wildlife to cope with stress derived from such interactions. Therefore, higher tolerance to stress can pose a significant adaptive advantage over animals that do not cope well with the stress caused by increased human contact. This hypothesis is in line with strong evidence of wildcat recovery and expansions across the structured anthropogenic habitats and mosaic-like landscapes [18, 19, 55] and other medium- and large-sized carnivores in Central Europe [56, 57].

### Introgression between wild and domestic cats, and its role on population divergence

We found some evidence of genome-wide introgression between German wildcats and domestic cats. However, the proportion of genome introgressed is relatively low, which supported previous evidence of low levels of introgression in German wildcats [21–23]. The relatively low levels of introgression observed in German wildcat populations might be due to the persistence of large forest patches in low mountainous regions [22], which may minimize both the opportunity and the necessity of interactions between wild and domestic congeners. In addition, we did not observe differential introgression for different wildcat populations. Deviations from the true bifurcating evolutionary history of the taxa are rather similar (Fig. 4b), indicating that gene flow between domestic cats and either Western or Central wildcats was and is equally likely, with no prevalence towards any of the groups. The chromosome level analysis, however, suggested a slightly increased level of introgression between Western wildcats and domestic cats (excess of ABBA sites (D > 1); Fig. 4a), which may be explained by the earlier contact of Western wildcats with domestic cats as the introduction of domestic cats likely happened first in south West Germany, which was part of the Roman Empire [27].

The relationship amongst haplotypes for each of the four highly differentiated genes reveals a distinct structure that corresponds to the different geographical groups defined in the analyses, a pattern which is neatly congruent with the population structure analyses (Figure S1, Supp. Information). Genetic drift, mutation and selection, but also by introgression from between congeners [58], shape the evolution of genomes in natural populations. However, in this case introgression from *FC* does not seem to play an important role in the differentiation of these genes between Western and Central wildcat populations, as shown by the very few haplotypes shared between domestic cat and the two wildcat populations. This might be due to the fact that introgression started relatively early in German populations and is generally very low [18, 21, 22], and overall, we do not see significant admixture in the four highly differentiated genes as it would be expected if introgression had contributed to population differentiation.

### Conservation implications

The observed ongoing spread of the European wildcat in Germany [18, 20] has been accompanied by large scale species conservation projects, aiming at facilitating the spread of the species by reducing habitat fragmentation within the patchy, mosaic like distribution of suitable habitats within the anthropogenically modified cultural landscape. For this, forest corridors were planted in different regions of the wildcat’s distribution to facilitate wildcats and other species disperse between isolated forest patches and migrate between different densely forested low mountain regions [59]. While these conservation attempts have gained considerable public attention, several related questions remain unanswered to date, such as potential negative effects of connectivity measures, e.g., related to potentially merging naturally isolated phylogenic lineages or if higher landscape connectivity may facilitate the spread of introgression from domestic cats.

While our findings may question the need for landscape migration corridors to facilitate gene flow between isolated habitats, they may still be valuable on a local perspective given the significant effects of and high abundance of effective landscape barriers, such as roads or settlements in the landscapes of Germany [60]. Given the recent anthropogenic divergence between west and central wildcat populations, there is currently no evidence against reconnecting them. Also, the relatively low proportion of introgression observed from domestic cats in the wildcat genomes indicated that an undesired spread of domestic cat genes into the wildcat population due to facilitated gene flow appears rather unlikely at the moment.

Given the fact that so far only a few corridors have been planned and at least partially implemented over rather short spatial distances [59], it appears unlikely that the observed high connectivity and genetic diversity as well as relatively low level of inbreeding detected in the wildcat genomes are consequences of those conservation actions. The observed patterns rather seem to be the consequence of the ongoing population increase and expansion after protection of the wildcat in the 1940s, as it has been documented in several studies [18–20, 61].

## Conclusions

Within Europe’s cultural landscapes, humans have had a major influence on wildlife for centuries, but the underlying genomic consequences of human-mediated activities on wildlife are still poorly understood. Although the use of population genomics applied to wildlife conservation is relatively new, it has been shown to have a great potential for the understanding of species ecology and biology as well as to inform species conservation management [11]. Our data suggest an evident impact of anthropogenic pressures on population differentiation and adaptive selection on German wildcat populations in the last 200 years. Future research should couple genomic evidence, such as the ones presented in this study, with behavioural evidence (e.g. identification of wildcat phenotypes that are more stress resistant) in order to test the hypotheses proposed above, and gain more insights on how short term anthropogenic activities and conservation strategies impact wildlife adaptive selection processes.

## Methods

### Sampling, sequencing, filtering and variant calling

**Fig. 5:**
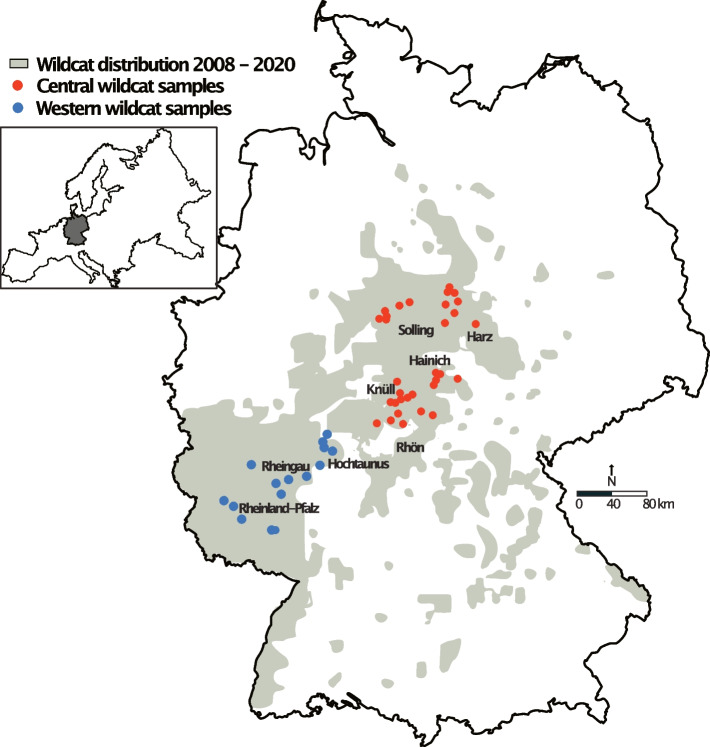
Wildcat sampling locations for this study. Red and blue dots represent central and western populations, respectively. Shaded grey area represents wildcat distribution between 2008 – 2020 according to Bund für Umwelt und Naturschutz Deutschland e.V. (BUND) -Wildkatzenwegeplan (www.wildkatzenwegeplan.de)

We whole-genome sequenced 47 *FS* individuals from across the species’ distribution in Germany (Fig. 5) and, 16 *FC* individuals from around the world. In addition, we obtained.fastq files for 21 domestic cat individuals from the ENA project PRJNA343389. Our final taxon sampling included 84 *Felis* individuals (Table S4 in Supp. Information). BGI Genomics conducted DNA isolation and library preparation. Sequencing was performed on a BGISEQ-500 platform, which generated 100 bp paired end reads per individual, and a mean coverage of 18-25X. Quality of raw whole genome sequence data of all 84 samples was checked using FastQC v0.11.5 [62] in combination with MultiQC v1.5 [63]. Quality checks indicated that reads were adapter free and were then mapped to the *Felis catus* v9.0 reference genome (European Nucleotide Archive (ENA) accession number: GCA_000181335) using BWA mem v0.7.15 [64] with default settings, and the *–M* option for Picard (Broad Institute 2019) compatibility (for downstream duplication marking. SNPs were called with Platypus v1.0 [65]. Bases with quality scores below 30 and reads with mapping quality below 30 were ignored, and only variants with at least 6 reads were kept.

We filtered for biallelic variants passing all filters and pruned all SNPs for Linkage disequilibrium (LD) with a squared correlation coefficient of more than 0.5 using bcftools v1.9 [66].

### Population structure analyses

We used ADMIXTURE version 1.3.0 [67] to infer ancestry proportions based on maximum likelihood estimations using an increasing number (*K*), from *K* = 1 to *K* = 15, with default settings and a tenfold cross validation procedure. We performed a second ADMIXTURE analysis for a subset that included only German wildcat individuals, *K* = 1 to *K* = 10 using the same settings as the first analysis. We subsequently performed a 10 runs analysis for *K* = 2, the *K* that received the highest support for German wildcat only. Results from both analyses were plotted with PONG [68]. In addition, we conducted a principal component analysis (PCA) on 1,899,534 unlinked SNPs on the two datasets, all *Felis* individuals and just German wildcat individuals, using the R package *Factoextra* v.1.0.7 [69].

### Demographic history inference

We implemented a pairwise sequentially Markovian coalescent (PSMC, [70]) model in order to reconstruct the demographic history of wildcat in Germany. We created.fastq sequences for eight unadmixed individuals selected based on ADMIXTURE results, including 2 of each of the following groups: western wildcats, central wildcats, western domestic cats and eastern domestic cats. We used SAMTOOLS v1.9 “mpileup” command [71] to filter for bases with a base quality of 30 and minimum coverage of SNPs of 10 as recommended by [72]. PSMC was run with 25 iterations and the upper limit of TMRCA was set to 5, initial ρ/θ value to 5 and *Ne* was inferred across 64 interval times (2*3 + 25*2 + 4 + 4) and calculated bootstrap ranges for each individual. Results were scaled with a mutation rate of µ = 1 × 10^–8^ per base pair and generation, assuming a generation time of two years [33]. We also implemented a multiple sequentially Markovian coalescence approach (MSMC2, [73] using the same set of individuals and groups as for the PSMC analysis). MSMC2 is a more advanced method which increases accuracy of the *Ne* history (especially inference of the recent past) and uses phased haplotype data of single chromosomes; therefore we separated each of the autosomal chromosomes and used Beagle v.5.4 [74] for phasing the genotypes using default parameters and window size of 10 kb.We used the generate_multihetsep.py (MSMC-TOOLS package; https://github.com/stschiff/msmc-tools) to generate the input files for MSMC2 for each chromosome. We used the same groups of unadmixed individuals, generation time, mutation rate and interval times as for the PSMC analysis. For the MSMC2 runs we used the option to skip sites with ambiguous phasing (-s) as recommended by instructions.

We performed a demographic modelling analysis where we defined four groups. Based on the clear population structuring from the ADMIXTURE analyses results we included central German *FS* and western German *FS*. The Middle East *FC* individuals were included in the analysis to polarize derived *vs* ancestral alleles, and the European *FC* as potential source of introgression with the *FS*. We designed four competing demographic models taking into account PSMC results and considering three criteria: 1) temporally different split time between central and western *FS*; 2) different temporally gene flow between European *FC*, central *FS* and western *FS*; and 3) different temporally introgression between European *FC* and *FS*. Based on these criteria we designed four models as follows: Model 1) depicts an early split of Western and Central German wildcat populations and differential introgression in wildcat; Model 2 depicts an early split of Western and Central German wildcat populations and late introgression; Model 3 depicts an late split of Western and Central German wildcat populations and early introgression; and Model 4 depicts an late split of Western and Central German wildcat populations and late introgression (Fig. 2). Here, we defined introgression as a one way admixture historical event (from domestic to wildcats). Detailed description of prior parameters for each model is given in the Supplementary Methods. We selected eight individuals per group based on GC% and coverage, except for near east *FC* for which just five samples were available. We conducted coalescent simulations of the multidimensional *site-frequency spectrum* (SFS) as summary statistics. In order to minimize the effects of selection on demographic inference, we included only neutrally and no linked evolving SNPs at fourfold degenerated sites in the genome by using tbg-tools v.0.2 (https://github.com/Croxa/tbg-tools) for selection. The selected SNPs were pruned for linkage disequilibrium (LD) with PLINK v 1.9 [75, 76], applying an r^2^ threshold of 0.1. We obtained the observed SFS of the unlinked, neutral SNPs using easySFS (https://github.com/isaacovercast/easySFS). We ran 10,000 simulations with fastsimcoal2 v.2.6.0.3 [77] for each model based on the observed multidimensional SFS (input files for the fastsimcoal2 analyses given in the Appendix). Simulated and observed SFS for each model were then compared using an Approximate Bayesian Computation (ABC) approach with the R package *abc* [78].

### ROH analyses

Runs of homozygosity (ROH) were identified using PLINK v 1.9 [75, 76]. We used the *--geno* option in PLINK before running the ROH analyses to keep only SNPs with a 90% genotyping rate (10% missing). There is a lack of consensus in the literature to whether SNP data should be pruned by LD and/or minor allele frequency (MAF). We followed Meyermans et al. [79] recommendations and skipped MAF pruning but tested different homozygosity window SNP and thresholds. In total we ran 3 analyses using the following options: 1) *–homozyg –homozyg-window-snp* 20 –*homozyg-window-het* 1 *–homozyg-window-missing* 5 *–homozyg-window-threshold* 0.05 –*homozyg-snp* 20 *–homozyg-het* 1 *–homozyg-density* 100 *–homozyg-kb* 1000 *–homozyg-gap* 1000; 2) we increased the *–homozyg-window-snp* to 50; and 3) *–homozyg-window-snp* 20 and *–homozyg-window-threshold* 0.25. We estimated the fraction of the genome in ROH (F_ROH_) as the total length of ROH per individual divided by the length of the genome. We evaluated significance level of the estimate using a pairwise t-test with Bonferroni correction.

### Fixation index analyses *F*_ST_

We estimated the genome-wide fixation index (*F*_ST_; [80]) for wildcat only (Western *vs* Central) using a non-overlapping 10-kb window with VCFtools 0.1.17 [81]. In order to get an indication of significant genes (outliers) under selection, we used parameters of the best-fit inferred demographic scenario to simulate 10-kb sequences using fastsimcoal2 v.2.6.0.3 [77] to infer *F*_ST_ distribution, under the assumption of no selection. We used a custom python script to extract genes for which any of the outliers 10-kb windows above the 99% quantile of the simulated *F*_ST_ distribution fall within the genomic coordinates of genes of the annotated genome of *FC*. Resulting genes were the candidates for functional enrichment analyses.

### Functional enrichment analyses

We ran a gene ontology (GO) term enrichment analysis on candidate genes that fell above the 99% *F*_ST_ threshold of simulated *F*_ST_. GO terms were annotated to all protein sequences using InterProScan v 5.39–77.0 [82], resulting in 45,297 GO terms which served as the reference for the functional enrichment analyses. We used 15,224 GO-annotated genes as universe. Analysis was carried out using the R package topGO v.2.42 [83] for the category biological processes (BP). We used the "weight01" algorithm and Fisher test for statistical support. We retained GO terms with a *p*-value ≤ 0.05 and used the Benjamini–Hochberg method [84] for *p*-value correction with a FDR (false discovery rate) level of 5%.

### Haplotype networks

In order to visualize and interpret individual relationships at the population level, we built haplotype networks, which can be more appropriate than hierarchical tree format for intraspecific analyses [85, 86]. We selected the four genes that corresponded to the 2 top GO terms from the enrichment analysis and extracted the DNA consensus haplotypes sequences from the *Felis catus* v9.0 reference genome for all cats included in our sampling using VCFtools 0.1.17 [81] and bcftools 2.4.37 [71]. Haplotype DNA sequences for all 84 individuals were phased with DnaSP v6 [87] and aligned using MAFFT v7 [88, 89]. We built haplotype networks for the four regions independently using the TCS algorithm [90] implemented in PopART 1.7 [91]. We coded individuals in 3 groups, corresponding to domestic cats, Central German wildcat and Western German wildcat.

### Introgression test

We tested for genome-wide introgression between wildcat and domestic cat with the ABBA-BABA test [92] using Patterson's *D*-statistic [93, 94]. We defined the groups as follows: P1 corresponds to Central wildcat, P2 corresponds to Western wildcat, P3 corresponds to European domestic cat. We used five Middle Eastern domestic cats as outgroup, and used the following species tree topology as null expectation: BBAA [(((P1,P2),P3),O)]. We tested 2 hypotheses to look at differential introgression as follows (Figure S8 in Supp. Information): 1) ABBA pattern would indicate gene flow between Western wildcat and domestic cat [(((P2,P3),P1),O)], and 2) BABA pattern gene flow between Central wildcat and domestic cat [(((P1,P3),P2),O)]. We calculated the genome-wide and chromosome level *D*-statistic using the genomics_general toolkit (https://github.com/simonhmartin/genomics_general; [92]) and the proportion of the genome introgressed (*f*). To test for the consistency of the estimation, we computed the standard deviation of *D*-statistic with the block jackknife procedure with 95% of the data, and the standard error, Z score and *p*-value for the test. In addition to this, we calculated *D*-statistic in 100-kb non-overlapping windows.

## Supplementary Information

**Additional file 1.** Supplementary Results, Supplementary Methods and Appendix A.

## Data Availability

Newly generated whole genome individual sequencing data is available at European Nucleotide Archive (ENA) project number: (to be added upon manuscript acceptance). fastq files for 21 domestic cat individuals can be found under ENA project PRJNA343389 (https://www.ebi.ac.uk/ena/browser/view/PRJNA343389).

## Abbreviations

ABC: Approximate Bayesian Computation
BP: Biological processes
CV-error: Cross validation error
FC: *Felis catus*
F_ROH_: Proportion of the genome contained in runs of homozygosity
FS: *Felis silvestris*
*F*_ST_: Fixation index
GO terms: Gene ontology terms
kya: Thousand years ago
MAF: Minor allele frequency
NAD: Nicotinamide
*N*_*e*_: Effective population size
PCA: Principal component analysis
PSMC: Pairwise sequentially Markovian coalescent
ROH: Runs of homozygosity
SFS: *site-frequency spectrum*
SNP: Single nucleotide polymorphism
TMRCA: Time to most recent common ancestor
WGS: Whole-genome sequencing
