# Correction: Human impact on the recent population history of the elusive European wildcat inferred from whole genome data

**DOI:** 10.1186/s12864-022-09047-w

**Published:** 2023-01-11

**Authors:** María Esther Nieto-Blázquez, Dennis Schreiber, Sarah A. Mueller, Katrin Koch, Carsten Nowak, Markus Pfenninger

**Affiliations:** 1 Molecular Ecology Group, Senckenberg Biodiversity and Climate Research Centre (SBiK-F), 60325 Frankfurt am Main, Germany; 2 Division of Evolutionary Biology, Faculty of Biology, Ludwig Maximilian University of Munich, Planegg-Martinsried, 82152 Munich, Germany; 3 Centre for Wildlife Genetics, Senckenberg Research Institute and Natural History Museum Frankfurt, 63571 Gelnhausen, Germany; 4 European Wildcat Monitoring, Bund Für Umwelt Und Naturschutz, 55118 Rheinland-Pfalz, Mainz Germany; 5 LOEWE Centre for Translational Biodiversity Genomics (LOEWE-TBG), 60325 Frankfurt am Main, Germany; 6 Institute for Molecular and Organismic Evolution, Johannes Gutenberg University, 55128 Mainz, Germany

**Correction: BMC Genomics 23, 709 (2022)**

**https://doi.org/10.1186/s12864-022-08930-w**

Following publication of the original article [1], the authors reported an error in the "Availability of data and materials" section, as they did not provide the European Nucleotide Archive (ENA) project number of the newly sequenced samples.

Therefore, the section should read as follows:

**Availability of data and materials**

Newly generated whole genome individual sequencing data is available at European Nucleotide Archive (ENA) project number: PRJEB40421 (https://www.ebi.ac.uk/ena/browser/view/PRJEB40421). fastq files for 21 domestic cat individuals can be found under ENA project PRJNA343389 (https://www.ebi.ac.uk/ena/browser/view/PRJNA343389).

The original article [1] has been updated.
