# Supplementary material for: Human impact on the recent population history of the elusive European wildcat inferred from whole genome data
Source: BMC Genomics. 2022 Oct 18;23:709. doi: 10.1186/s12864-022-08930-w (PMC9578205; doi:10.1186/s12864-022-08930-w)
Supplement: Supplementary file 1 — Additional file 1. Supplementary Results, Supplementary Methods and Appendix A. [file 12864_2022_8930_MOESM1_ESM.docx]

**Supporting Information**

**TITLE Human impact on the recent population history of the elusive European wildcat inferred from whole genome data**

**María Esther Nieto-Blázquez^1^**, Dennis Schreiber^1^, **Sarah Mueller**^2^**,** [**Katrin Koch**](https://office.mailbox.org/appsuite/)**^3^, Carsten Nowak^4,5^,** and Markus Pfenninger^1,5,6^

*^1^Department of Molecular Ecology, Senckenberg Biodiversity and Climate Research Centre, Frankfurt am Main 60325, Germany*

*^2^Division of Evolutionary Biology, Faculty of Biology, Ludwig Maximilian University of Munich, Planegg-Martinsried 82152, Germany*

*^3^European Wildcat Monitoring, Bund für Umwelt und Naturschutz, Rheinland-Pfalz, 55118 Mainz, Germany*

*^4^Centre for Wildlife Genetics, Senckenberg Research Institute and Natural History Museum Frankfurt, Gelnhausen 63571, Germany

^5^LOEWE Centre for Translational Biodiversity Genomics (LOEWE-TBG), Frankfurt am Main 60325, Germany*

*^6^Institute for Molecular and Organismic Evolution, Johannes Gutenberg University, Mainz 55128, Germany*

**Supplementary Results**

- Table S1 - Enriched GO terms for Biological processes …………….……………. p. 2
- Table S2 – ABBA-BABA chromosome level *D*-statistics ………………………... p.3
- Table S3 - Parameter estimates for best model in fastsimcoal …………….…….... p. 3
- Figure S1 - ADMIXTURE results for domestic and wildcat……………………… p. 4
- Figure S2 – PSMC and MSMC2 plots ……………………………………………. p. 5
- Figure S3 – PSMC bootstrap ranges for each individual. ………………………… p. 6
- Figure S4 - Histogram showing the good fit of Model 4 to the data ………....…......p. 7
- Figure S5 - FROH and ROHs length per individual………………………….…… p. 8
- Figure S6 – Sketch of tryptophan-kynurenine-serotonin pathway ……...………… p. 9
- Figure S7 - Haplotype networks for the four differentiated genes ……………...… p. 10

**Supplementary Methods**

- Table S3 - Sampling individual information …………....…………………….….. p. 12
- Description of demographic scenarios simulated in fastsimcoal2 ………...……… p. 15
- Figure S8 - Sketch of ABBA-BABA hypotheses tested …………………..……… p. 15

**Appendix A:** *fastsimcoal2* model definition input files ……………………………….….. p. 16

**Supplementary Results**

**Table S1.** Parameter estimates for best model (Model 4). All Ns are in number of haploid individuals. T = time; ANC = ancestral; MEc = Middle Eastern *Felis catus*; Ec = European *Felis catus*; Ws = Western *Felis* *silvestris*; and Cs = Central *Felis* *silvestris*.

| **Parameter** | **Estimated value** | **95% CI lower bound** | **95% CI upper bound** |
| --- | --- | --- | --- |
| NANC | 58491 | 52836 | 64146 |
| TDIV | 363455 | 301743 | 425167 |
| TIME1 | 10393 | 9974 | 10812 |
| TIME2 | 869 | 742 | 996 |
| TBOT | 504 | 422 | 586 |
| TENDBOT | 404 | 325 | 483 |
| TIME3 | 388 | 220 | 556 |
| N1_ANC | 80522 | 65287 | 95757 |
| N2_ANC | 52022 | 29768 | 74276 |
| NENDBOT | 47859 | 29127 | 66591 |
| NBOT | 93315 | 56622 | 130008 |
| NPOP0 | 76087 | 64246 | 87928 |
| NPOP1 | 93821 | 77643 | 109999 |
| NPOP2 | 83518 | 79488 | 87548 |
| NPOP3 | 63922 | 38374 | 89470 |

**
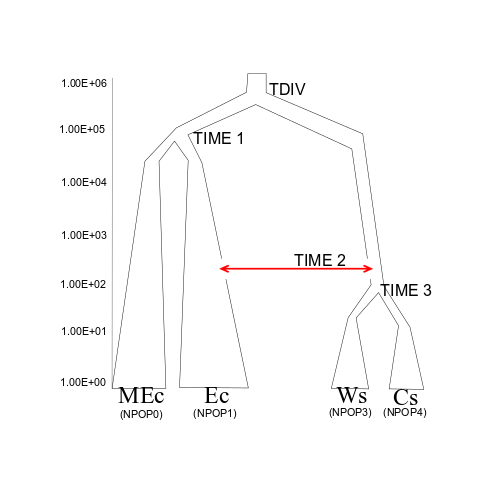
**

**Table S2.** Gene Ontology (GO) term enrichment analysis results for the genes above the 99% quantile of the simulated *F*_ST_. Significantly (P < 0.05) overrepresented GO terms in the category biological process (BP).

|  | **GO ID** | **Term** | **Annotated** | **Significant** | **Expected** | **Fisher’s exact test *p*-value** | **FDR corrected *p*-value** |
| --- | --- | --- | --- | --- | --- | --- | --- |
| 1 | GO:0070189***** | kynurenine metabolic process | 6 | 2 | 0.02 | 0.00023 | 0.03312 |
| 2 | GO:0006569***** | tryptophan catabolic process | 6 | 2 | 0.02 | 0.00023 | 0.03312 |
| 3 | GO:0006139 | nucleobase-containing compound metabolic... | 2194 | 8 | 8.75 | 0.00211 | 0.22816 |
| 4 | GO:0006903 | vesicle targeting | 5 | 1 | 0.02 | 0.0198 | 0.8084290 |
| 5 | GO:1905515 | non-motile cilium assembly | 5 | 1 | 0.02 | 0.0198 | 0.8084290 |
| 6 | GO:0006265 | DNA topological change | 5 | 1 | 0.02 | 0.0198 | 0.8084290 |
| 7 | GO:0005978 | glycogen biosynthetic process | 6 | 1 | 0.02 | 0.02371 | 0.8084290 |
| 8 | GO:0006188 | IMP biosynthetic process | 6 | 1 | 0.02 | 0.02371 | 0.8084290 |
| 9 | GO:0006488 | dolichol-linked oligosaccharide biosynth... | 7 | 1 | 0.03 | 0.02761 | 0.8084290 |
| 10 | GO:0043101 | purine-containing compound salvage | 8 | 1 | 0.03 | 0.03149 | 0.8084290 |
| 11 | GO:0007052 | mitotic spindle organization | 8 | 1 | 0.03 | 0.03149 | 0.8084290 |
| 12 | GO:0032259 | methylation | 71 | 2 | 0.28 | 0.0347 | 0.81673 |
| 13 | GO:0007205 | protein kinase C-activating G protein-co... | 10 | 1 | 0.04 | 0.03921 | 0.8271375 |
| 14 | GO:0017004 | cytochrome complex assembly | 11 | 1 | 0.04 | 0.04305 | 0.8271375 |
| 15 | GO:0006904 | vesicle docking involved in exocytosis | 12 | 1 | 0.05 | 0.04687 | 0.8271375 |
| 16 | GO:0009966 | regulation of signal transduction | 207 | 3 | 0.83 | 0.04903 | 0.8271375 |

***** See below ID’s of the genes annotated to the two significant GO terms:

- GO:0070189: gene ID’s 101082605 and 101082171

- GO:0006569: gene ID’s 101095789 and 101096086

**Table S3.** ABBA-BABA chromosome level *D*-statistics. Chromosomes with no significant estimation (Z-score < 3-4) indicated by *.

| **Chromosome** | ***D*-statistic mean** | **Variance** | **Standard deviation** | **Standard error** | **Z-score** |
| --- | --- | --- | --- | --- | --- |
| 1 | 0.04119199 | 0.01287405 | 0.1134639 | 0.007293733 | 5.647587 |
| 2 | 0.0517333 | 0.01273561 | 0.1128522 | 0.00863002 | 5.994574 |
| 3 | 0.05254425 | 0.009729043 | 0.09863591 | 0.008248349 | 6.370275 |
| 4 | 0.04313738 | 0.009844396 | 0.09921893 | 0.006879595 | 6.270337 |
| 5 | 0.08167795 | 0.01089036 | 0.1043569 | 0.008382148 | 9.744274 |
| 6 | 0.0479054 | 0.01177636 | 0.1085189 | 0.008890218 | 5.388552 |
| 7 | 0.03656435 | 0.009600795 | 0.09798365 | 0.008165304 | 4.478015 |
| 8 | 0.05550572 | 0.009361785 | 0.09675632 | 0.006493859 | 8.547417 |
| 9 | 0.05993018 | 0.007295844 | 0.08541571 | 0.006731701 | 8.902679 |
| 10 | 0.05610697 | 0.0068498 | 0.08276352 | 0.00765149 | 7.332816 |
| 11 | 0.05977754 | 0.01101087 | 0.1049327 | 0.01106087 | 5.404414 |
| 12 | 0.05493681 | 0.008154521 | 0.09030239 | 0.009216449 | 5.960735 |
| 13 | 0.06696098 | 0.01673758 | 0.1293738 | 0.01320416 | 5.071204 |
| 14 | 0.07464102 | 0.01057496 | 0.1028346 | 0.01295594 | 5.761141 |
| 15 | 0.07547414 | 0.01344032 | 0.1159324 | 0.01449155 | 5.208148 |
| 16 | 0.06906134 | 0.009667346 | 0.09832266 | 0.01499407 | 4.605912 |
| 17* | 0.02408104 | 0.008898303 | 0.09433082 | 0.01127469 | 2.135849 |
| 18* | 0.03180567 | 0.01385245 | 0.1176964 | 0.01284173 | 2.476743 |
| 19* | 0.04469147 | 0.05135805 | 0.2266231 | 0.01987616 | 2.248496 |

**Figure S1.** ADMIXTURE population structure plots for domestic and wildcat (*K*=1 to *K*=10).

**
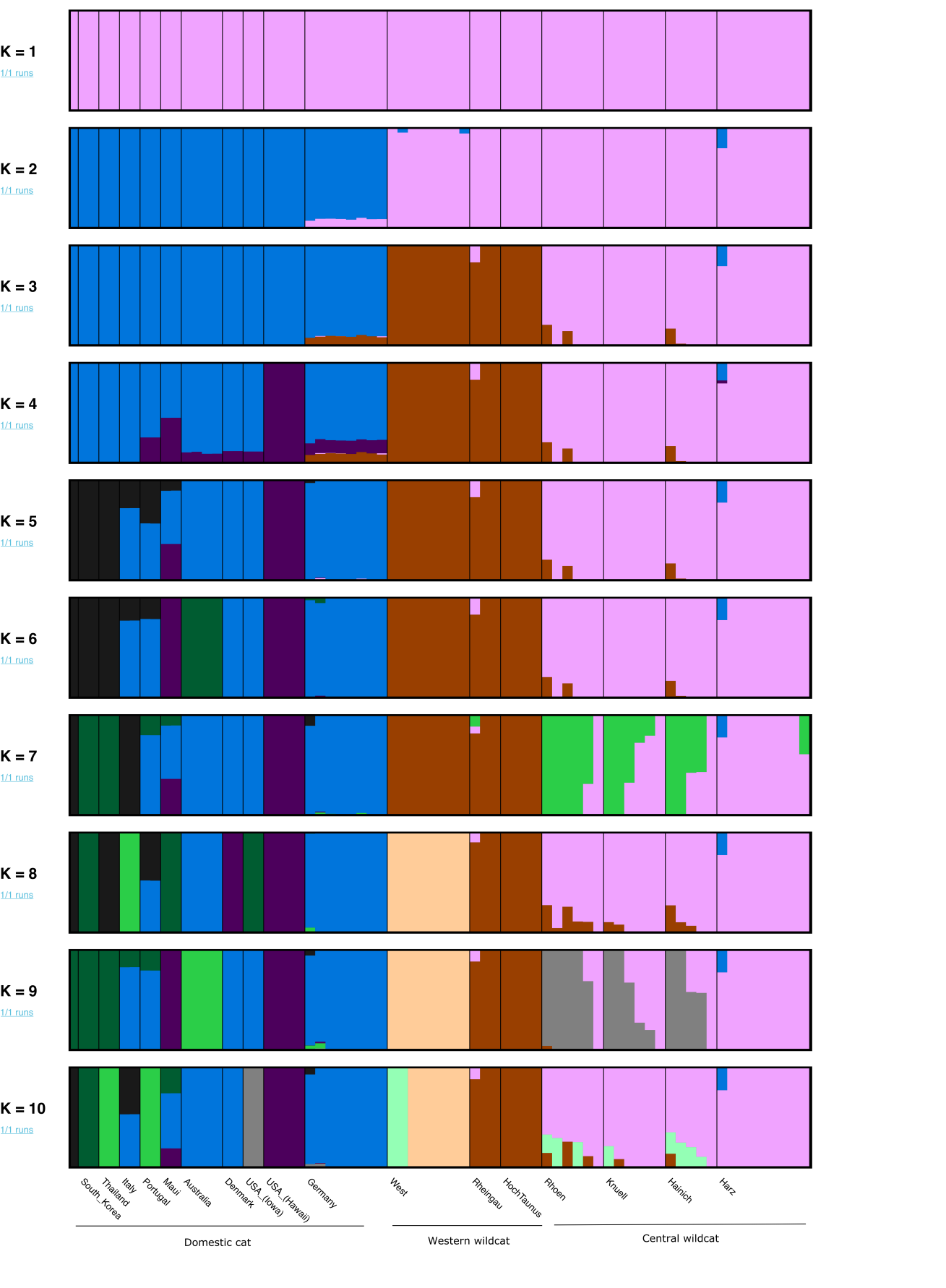
**

**Figure S2.** (a) PSMC plot corresponding to the historical effective population size (*N_e_*) for domestic (shades of blue) and wild cat (shades of brown). WC = wildcat, DC = domestic cat, g = generation time (in years), and µ = mutation rate; (b) MSMC2 plot corresponding to the historical effective population size (*N_e_*) for domestic (green and light blue blue) and wild cat (blue and red).

**
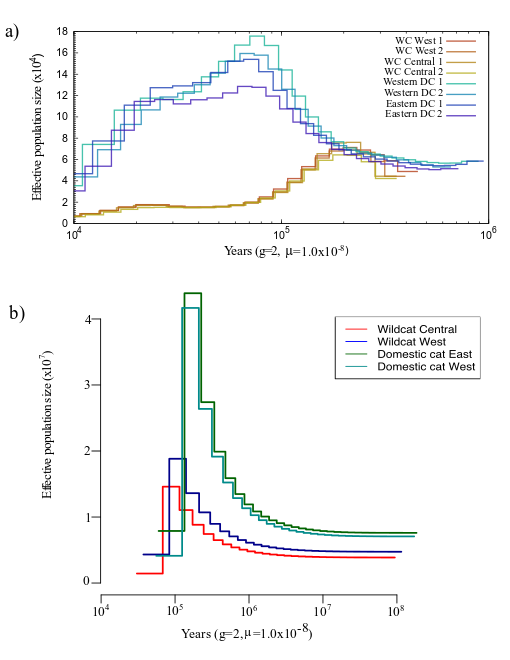
**

**Figure S3.** Bootstrap confidence ranges for each of the individuals included in the PSMC analysis.

**
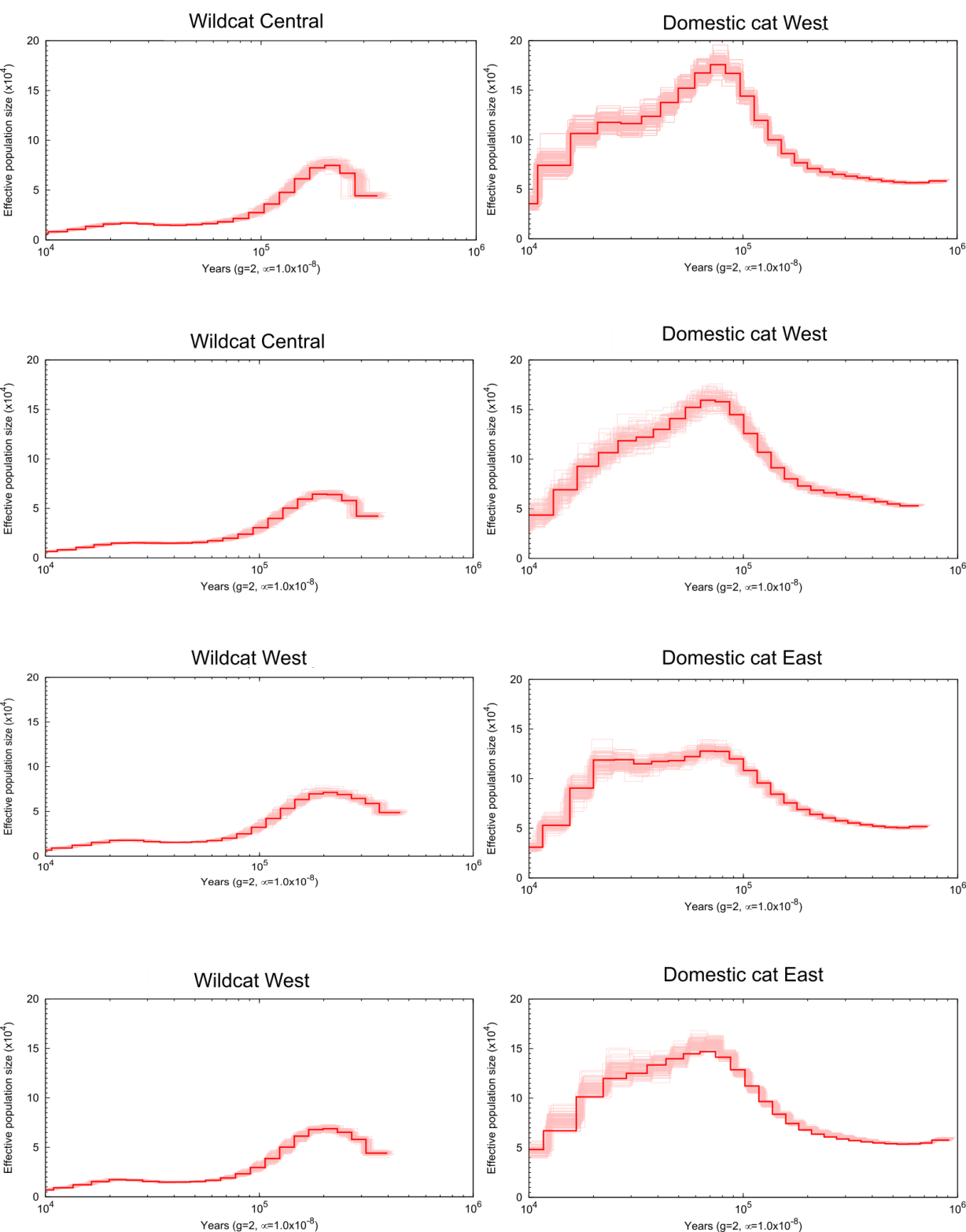
**

**Figure S4.** Histogram showing the good fit of Model 4 to the data.

**
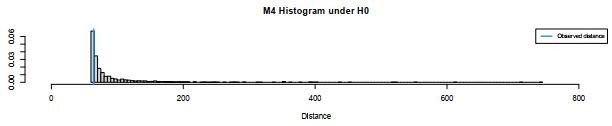
**

**Figure S5**. (a) Proportion of the genome that was contained in runs of homozygosity (FROH). (b) Bar plot showing ROHs length per individual.


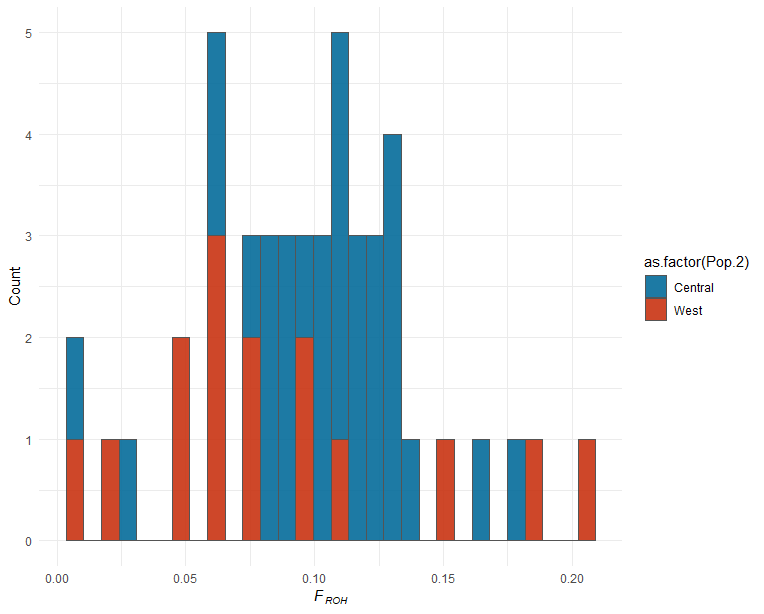
a)


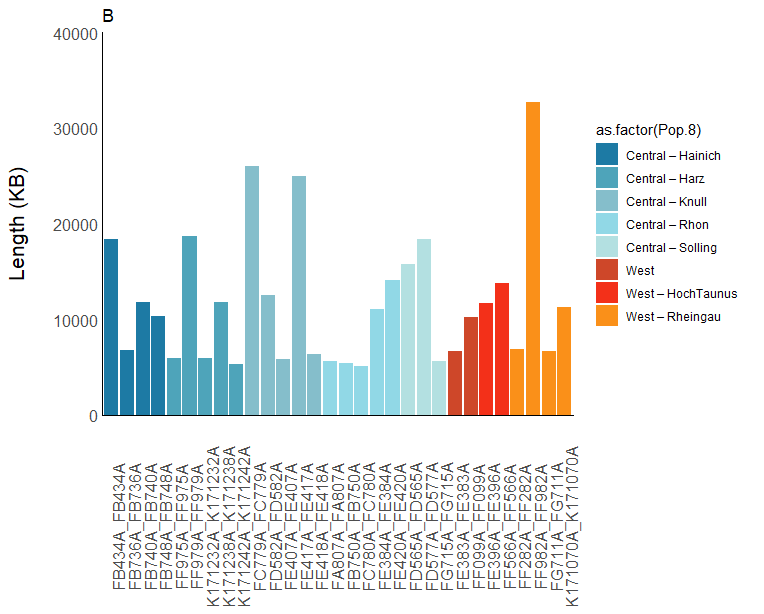


b)

**Figure S6.** Tryptophan-kynurenine-serotonin pathway simplified sketch. Numbers correspond to EC (enzyme) ids as in UniProt.


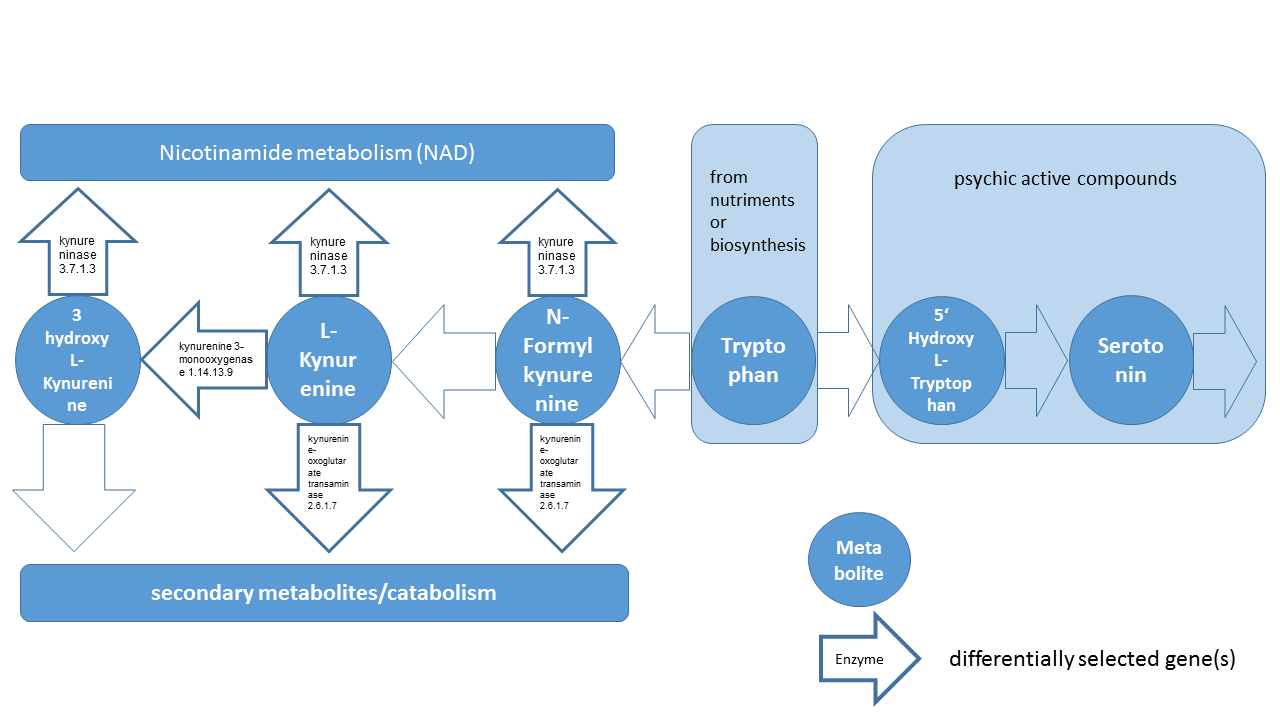


**Figure S7**. Haplotype networks for the four highly differentiated genes obtained in the GO term enrichment analyses.

a)


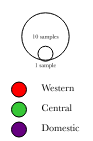

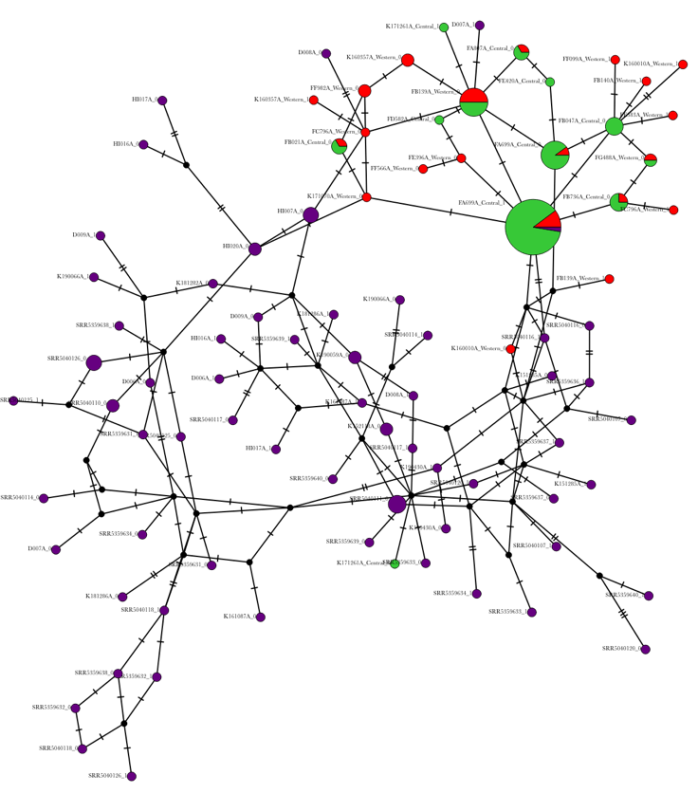


b)


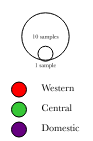
**
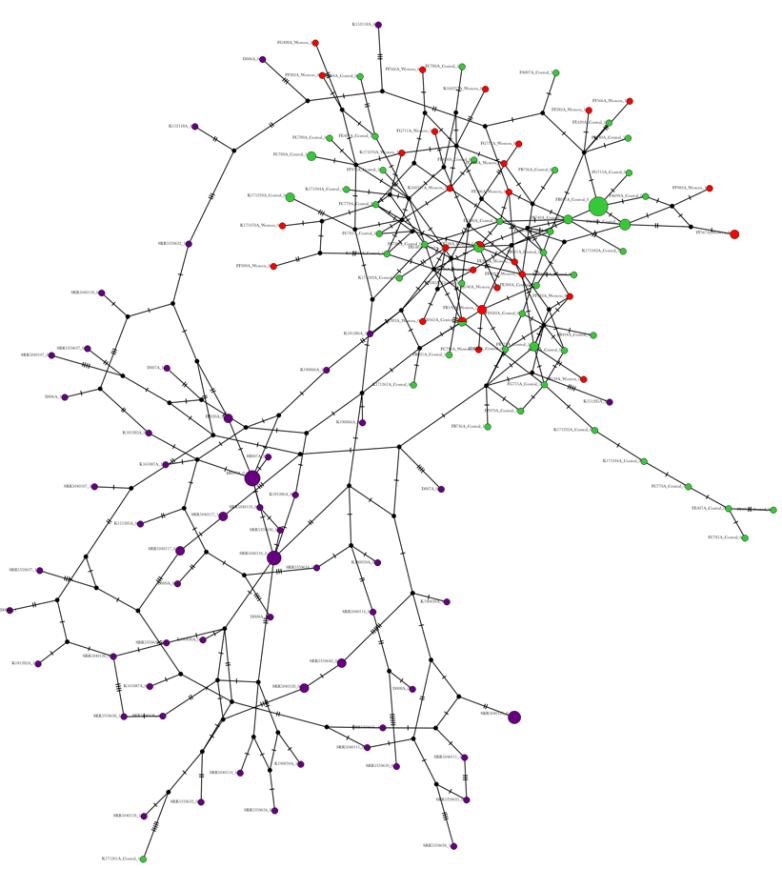
**

c)


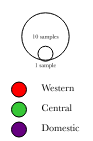
**
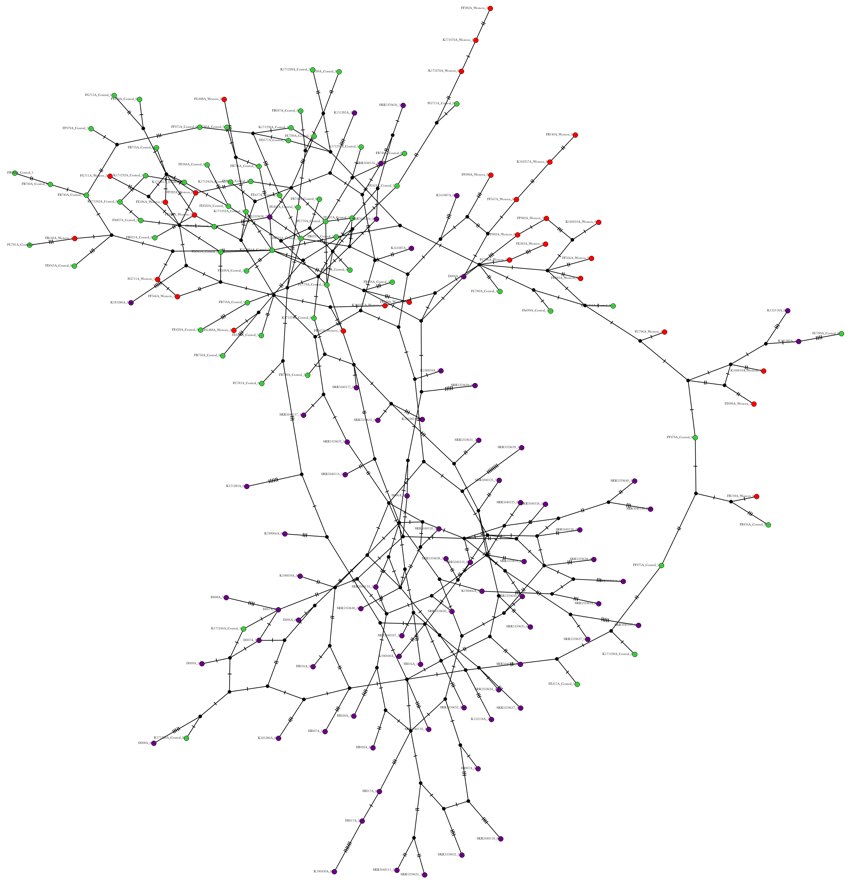
**

d)


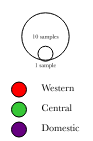
**
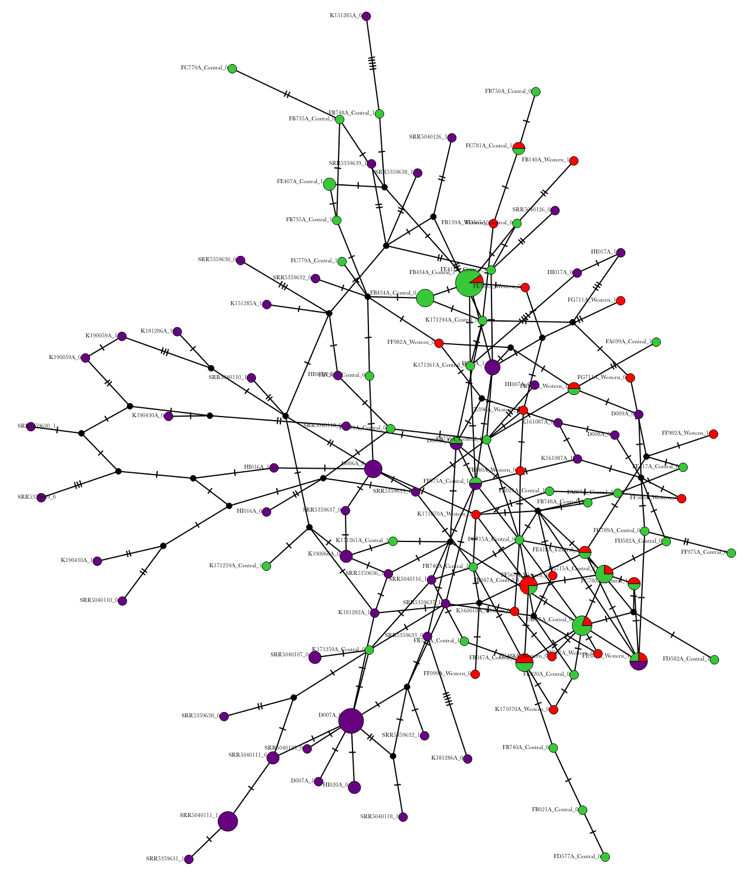
**

**Supplementary Methods**

**Table S4.** Individual’s sampling location information. C = Central wildcat, W = Western wildcat. Individuals in bold correspond to previously sequenced data from ENA project PRJNA343389.

| **Wild cat (*Felis* *silvestris*)** | | | | | **Domestic cat (*Felis catus*)** | |
| --- | --- | --- | --- | --- | --- | --- |
| **Individual ID** | **Group** | **Population** | **Latitude (N)** | **Longitude (E)** | **Individual ID** | **Origin** |
| FA699 | C | Solling | 51.82859 | 9.82147 | D006A | Australia |
| FG715 | C | Solling | 51.643589 | 9.45638 | D007A | Australia |
| FB021 | C | Solling | 51.75285 | 9.4639 | D008A | Australia |
| FB047 | C | Solling | 51.638889 | 9.37811 | D009A | Australia |
| FD565 | C | Solling | 51.792381 | 9.68892 | K151285A | Germany |
| FD577 | C | Solling | 51.653721 | 9.47219 | K152118A | Germany |
| FB434 | C | Hainich | 51.055481 | 10.57035 | K161087A | Germany |
| FB735 | C | Hainich | 51.018421 | 10.24262 | K181282A | Germany |
| FB736 | C | Hainich | 51.125671 | 10.27347 | K181286A | Germany |
| FB740 | C | Hainich | 51.061909 | 10.26109 | K190059A | Germany |
| FB748 | C | Hainich | 51.098358 | 10.33074 | K190066A | Germany |
| FD582 | C | Knüll | 50.803551 | 9.53809 | K190430A | Germany |
| FC779 | C | Knüll | 51.001438 | 9.6456 | HI007A | USA (Hawaii) |
| FC781 | C | Knüll | 50.899139 | 9.86611 | HI016A | USA (Hawaii) |
| FE407 | C | Knüll | 50.851879 | 9.69569 | HI017A | USA (Hawaii) |
| FE417 | C | Knüll | 50.888939 | 9.82877 | HI020A | USA (Hawaii) |
| FE418 | C | Knüll | 50.848549 | 9.64058 | **SRR5359632** | **Jordan** |
| FE384 | C | Rhön | 50.595379 | 9.30966 | **SRR5040111** | **Madagascar** |
| FC789 | C | Rhön | 50.73848 | 9.68969 | **SRR5359635** | **Madagascar** |
| FE420 | C | Rhön | 50.684689 | 9.65354 | **SRR5040125** | **Maui** |
| FA807 | C | Rhön | 50.691319 | 10.22264 | **SRR5359631** | **Maui** |
| FB750 | C | Rhön | 50.728889 | 10.02548 | **SRR5040107** | **Oman** |
| FC780 | C | Rhön | 50.603149 | 9.7291 | **SRR5359637** | **Oman** |
| FF975 | C | Harz | 51.862389 | 10.61854 | **SRR5040114** | **Portugal** |
| FF979 | C | Harz | 51.600811 | 10.8918 | **SRR5359634** | **Portugal** |
| K171228 | C | Harz | 51.94696 | 10.51846 | **SRR5040126** | **South Korea** |
| K171232 | C | Harz | 51.800159 | 10.45175 | **SRR5359638** | **South Korea** |
| K171238 | C | Harz | 51.71611 | 10.56326 | **SRR5040120** | **Thailand** |
| K171242 | C | Harz | 51.92165 | 10.55705 | **SRR5359640** | **Thailand** |
| K171244 | C | Harz | 51.92165 | 10.55705 | **SRR5040110** | **USA (Iowa)** |
| K171259 | C | Harz | 51.62344 | 10.42955 | **SRR5359630** | **USA (Iowa)** |
| K171261 | C | Harz | 51.92062 | 10.5168 | **SRR5040116** | **Denmark** |
| FB139 | W | Rheinland-Pfalz | 49.77927 | 6.84335 | **SRR5359636** | **Denmark** |
| FB140 | W | Rheinland-Pfalz | 49.584251 | 7.10679 | **SRR5359639** | **Iraq** |
| FC796 | W | Rheinland-Pfalz | 50.185169 | 7.29749 | **SRR5040117** | **Italy** |
| FE383 | W | Rheinland-Pfalz | 49.984089 | 7.70514 | **SRR5359633** | **Italy** |
| FF099 | W | Rheinland-Pfalz | 49.7085 | 6.9866 | **SRR5040118** | **Jordan** |
| K160010 | W | Rheinland-Pfalz | 49.477791 | 7.64843 |  |  |
| K160357 | W | Rheinland-Pfalz | 49.474529 | 7.62236 |  |  |
| FG488 | W | Rheinland-Pfalz | 49.867321 | 7.66957 |  |  |
| FF566 | W | Hochtaunus | 50.387989 | 8.45735 |  |  |
| FF567 | W | Hochtaunus | 50.31778 | 8.46556 |  |  |
| FF982 | W | Hochtaunus | 50.302761 | 8.56787 |  |  |
| FE396 | W | Hochtaunus | 50.459759 | 8.51585 |  |  |
| FG711 | W | Rheingau-Taunus | 50.037029 | 8.15946 |  |  |
| FF282 | W | Rheingau-Taunus | 49.99448 | 7.90611 |  |  |
| K171070 | W | Rheingau-Taunus | 50.144032 | 8.36563 |  |  |

**Description of demographic scenarios simulated in fastsimcoal2**

We tested four competing demographic scenarios which we designed based on the following criteria: 1) temporally different split time between central and western *FS*; 2) different temporally gene flow between European *FC*, central *FS* and western *FS*; and 3) different temporally introgression between European *FC* and *FS* .

All effective population sizes (*N_e_* values) were set to a uniform distribution and values of 4e^4^ (min) and 1e^5^ (max) in number of individuals. We chose these values based on the PSMC analysis, which suggested a peak on *N_e_* values for the wildcat of. 7e^4^ individuals). Migration rates were set to a log-uniform distribution and values of 1e^-10^ (min) and 1e^-3^ (max).

All times for the different historical events were set to uniform distribution. Historical events values were set as follows:

- Model 1 depicts an early split (10,000-30,000 generations ago) of Western and Central German wildcat populations and differential introgression in wildcat.
- Model 2 depicts an early split (10,000-30,000 generations ago) of Western and Central German wildcat populations and
- Model 3 depicts a late split (200-400 generations ago) of Western and Central German wildcat populations and early (4000-5000 generations ago) introgression.
- Model 4 depicts a late split (200-400 generations ago) wildcat populations and late (700-1000 generations ago) introgression.

**Figure S8.** ABBA-BABA analyses hypotheses tested. (a) Indicates null expectation; (b) indicates ABBA pattern; and (c) indicates BABA pattern. Ws = Western Germany wildcat; and Cs = Central Germany wildcat; Ec = European domestic cat; and Outg. = outgroup. Red arrow indicates introgression.

**
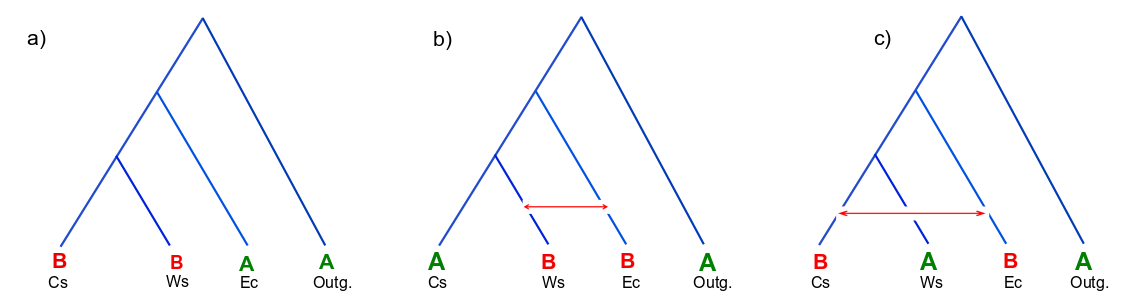
**

**Appendix A:** *fastsimcoal2* model definition input files (.est and .tpl files) for the 4 models tested.

Scenario1.est

// Search ranges and rules file

// ****************************

//$NPOP0$

//$NPOP1$

//$NPOP2$

//$NPOP3$

[PARAMETERS]

//#isInt? #name #dist.#min #max

//all Ns are in number of haploid individuals

1 $TDIV$ unif 300000 400000 output

1 $TIME1$ unif 20000 40000 output

1 $TIME2$ unif 10000 30000 output

1 $TIME3$ unif 700 900 output

1 $TBOT_1$ unif 90 120 output

1 $TENDBOT_1$ unif 70 90 output

1 $TBOT_2$ unif 90 120 output

1 $TENDBOT_2$ unif 70 90 output

1 $NANC$ unif 40000 100000 output

1 $N1_ANC$ unif 40000 100000 output

1 $N2_ANC$ unif 40000 100000 output

1 $NANC_POP1$ unif 40000 100000 output

1 $NANC_POP2$ unif 40000 100000 output

1 $NENDBOT_1$ unif 40000 100000 output

1 $NBOT_1$ unif 40000 100000 output

1 $NENDBOT_2$ unif 40000 100000 output

1 $NBOT_2$ unif 40000 100000 output

1 $NPOP0$ unif 40000 100000 output

1 $NPOP1$ unif 40000 100000 output

1 $NPOP2$ unif 40000 100000 output

1 $NPOP3$ unif 40000 100000 output

0 $Mig12$ logunif 1e-10 1e-3 output

0 $Mig13$ logunif 1e-10 1e-3 output

[RULES]

1 $TDIV$ > $TIME1$

1 $TIME1$ > $TIME2$

1 $TIME2$ > $TIME3$

1 $TIME3$ > $TBOT_1$

1 $TIME3$ > $TBOT_2$

1 $TBOT_1$ > $TENDBOT_1$

1 $TBOT_2$ > $TENDBOT_2$

[COMPLEX PARAMETERS]

0 $RESIZE$ = $NANC$/$N1_ANC$ hide

0 $RESIZE1$ = $N1_ANC$/$NPOP0$ hide

0 $RESIZE2$ = $N2_ANC$/$NANC_POP1$ hide

0 $RESIZE3$ = $NANC_POP1$/$NBOT_1$ hide

0 $RESIZE4$ = $NANC_POP2$/$NBOT_2$ hide

0 $RESIZE5$ = $NBOT_1$/$NENDBOT_1$ hide

0 $RESIZE6$ = $NBOT_2$/$NENDBOT_2$ hide

Scenario1.tpl

//Parameters for the coalescence simulation program : simcoal.exe

4 samples to simulate :

//Population effective sizes (number of genes)

$NPOP0$

$NPOP1$

$NPOP2$

$NPOP3$

//Samples sizes and samples age

5

8

8

8

//Growth rates: negative growth implies population expansion

0

0

0

0

//Number of migration matrices : 0 implies no migration between demes

3

//Migration matrix 0

0 0 0 0

0 0 0 0

0 0 0 0

0 0 0 0

//Migration matrix 1

0 0 0 0

0 0 0 $Mig13$

0 0 0 0

0 0 0 0

//Migration matrix 2

0 0 0 0

0 0 $Mig12$ 0

0 0 0 0

0 0 0 0

//historical event: time, source, sink, migrants, new deme size, growth rate, migr mat index

9 historical event

$TENDBOT_2$ 3 3 0 $RESIZE6$ 0 0

$TENDBOT_1$ 2 2 0 $RESIZE5$ 0 0

$TBOT_2$ 3 3 0 $RESIZE4$ 0 0

$TBOT_1$ 2 2 0 $RESIZE3$ 0 0

$TIME3$ 1 1 1 1 0 2

$TIME2$ 1 1 1 1 0 1

$TIME1$ 2 3 1 $RESIZE2$ 0 0

$TIME1$ 0 1 1 $RESIZE1$ 0 0

$TDIV$ 1 3 1 $RESIZE$ 0 0

//Number of independent loci [chromosome]

1 0

//Per chromosome: Number of contiguous linkage Block: a block is a set of contiguous loci

1

//per Block:data type, number of loci, per gen recomb and mut rates

SNP 4410 0 1e-8

============================================================

============================================================

Scenario2.est

// Search ranges and rules file

// ****************************

//$NPOP0$

//$NPOP1$

//$NPOP2$

//$NPOP3$

[PARAMETERS]

//#isInt? #name #dist.#min #max

//all Ns are in number of haploid individuals

1 $TDIV$ unif 300000 400000 output

1 $TIME1$ unif 20000 40000 output

1 $TIME2$ unif 10000 30000 output

1 $TBOT_1$ unif 90 120 output

1 $TENDBOT_1$ unif 70 90 output

1 $TBOT_2$ unif 90 120 output

1 $TENDBOT_2$ unif 70 90 output

1 $NANC$ unif 40000 100000 output

1 $N1_ANC$ unif 40000 100000 output

1 $N2_ANC$ unif 40000 100000 output

1 $NANC_POP1$ unif 40000 100000 output

1 $NANC_POP2$ unif 40000 100000 output

1 $NENDBOT_1$ unif 40000 100000 output

1 $NBOT_1$ unif 40000 100000 output

1 $NENDBOT_2$ unif 40000 100000 output

1 $NBOT_2$ unif 40000 100000 output

1 $NPOP0$ unif 40000 100000 output

1 $NPOP1$ unif 40000 100000 output

1 $NPOP2$ unif 40000 100000 output

1 $NPOP3$ unif 40000 100000 output

0 $Mig12$ logunif 1e-10 1e-3 output

0 $Mig13$ logunif 1e-10 1e-3 output

[RULES]

1 $TDIV$ > $TIME1$

1 $TIME1$ > $TIME2$

1 $TIME2$ > $TBOT_1$

1 $TIME2$ > $TBOT_2$

1 $TBOT_1$ > $TENDBOT_1$

1 $TBOT_2$ > $TENDBOT_2$

1 $NPOP2$ > $NPOP3$

[COMPLEX PARAMETERS]

0 $RESIZE$ = $NANC$/$N1_ANC$ hide

0 $RESIZE1$ = $N1_ANC$/$NPOP0$ hide

0 $RESIZE2$ = $N2_ANC$/$NANC_POP1$ hide

0 $RESIZE3$ = $NANC_POP1$/$NBOT_1$ hide

0 $RESIZE4$ = $NANC_POP2$/$NBOT_2$ hide

0 $RESIZE5$ = $NBOT_1$/$NENDBOT_1$ hide

0 $RESIZE6$ = $NBOT_2$/$NENDBOT_2$ hide

Scenario2.tpl

//Parameters for the coalescence simulation program : simcoal.exe

4 samples to simulate :

//Population effective sizes (number of genes)

$NPOP0$

$NPOP1$

$NPOP2$

$NPOP3$

//Samples sizes and samples age

5

8

8

8

//Growth rates: negative growth implies population expansion

0

0

0

0

//Number of migration matrices : 0 implies no migration between demes

2

//Migration matrix 0

0 0 0 0

0 0 0 0

0 0 0 0

0 0 0 0

//Migration matrix 1

0 0 0 0

0 0 $Mig12$ $Mig13$

0 0 0 0

0 0 0 0

//historical event: time, source, sink, migrants, new deme size, growth rate, migr mat index

8 historical event

$TENDBOT_2$ 3 3 0 $RESIZE6$ 0 0

$TENDBOT_1$ 2 2 0 $RESIZE5$ 0 0

$TBOT_2$ 3 3 0 $RESIZE4$ 0 0

$TBOT_1$ 2 2 0 $RESIZE3$ 0 0

$TIME2$ 1 1 1 1 0 1

$TIME1$ 2 3 1 $RESIZE2$ 0 0

$TIME1$ 0 1 1 $RESIZE1$ 0 0

$TDIV$ 1 3 1 $RESIZE$ 0 0

//Number of independent loci [chromosome]

1 0

//Per chromosome: Number of contiguous linkage Block: a block is a set of contiguous loci

1

//per Block:data type, number of loci, per gen recomb and mut rates

SNP 4410 0 1e-8

============================================================

============================================================

Scenario3.est

// Search ranges and rules file

// ****************************

//$NPOP0$

//$NPOP1$

//$NPOP2$

//$NPOP3$

[PARAMETERS]

//#isInt? #name #dist.#min #max

//all Ns are in number of haploid individuals

1 $NANC$ unif 40000 100000 output

1 $TDIV$ unif 300000 400000 output

1 $TIME1$ unif 9000 11000 output

1 $TIME2$ unif 4000 5000 output

1 $TBOT$ unif 500 600 output

1 $TENDBOT$ unif 400 500 output

1 $TIME3$ unif 200 400 output

1 $N1_ANC$ unif 40000 100000 output

1 $N2_ANC$ unif 40000 100000 output

1 $NENDBOT$ unif 40000 100000 output

1 $NBOT$ unif 40000 100000 output

1 $NPOP0$ unif 40000 100000 output

1 $NPOP1$ unif 40000 100000 output

1 $NPOP2$ unif 40000 100000 output

1 $NPOP3$ unif 40000 100000 output

0 $MigA$ logunif 1e-10 1e-3 output

[RULES]

1 $TDIV$ > $TIME1$

1 $TIME1$ > $TIME2$

1 $TIME2$ > $TBOT$

1 $TENDBOT$ > $TIME3$

1 $NENDBOT$ < $NPOP2$

1 $NENDBOT$ < $NPOP3$

1 $NPOP2$ > $NPOP3$

[COMPLEX PARAMETERS]

0 $RESIZE$ = $NANC$/$N1_ANC$ hide

0 $RESIZE1$ = $N1_ANC$/$NPOP0$ hide

0 $RESIZE2$ = $N2_ANC$/$NBOT$ hide

0 $RESIZE3$ = $NBOT$/$NENDBOT$ hide

0 $RESIZE4$ = $NENDBOT$/$NPOP2$ hide

Scenario3.tpl

//Parameters for the coalescence simulation program : simcoal.exe

4 samples to simulate :

//Population effective sizes (number of genes)

$NPOP0$

$NPOP1$

$NPOP2$

$NPOP3$

//Samples sizes and samples age

5

8

8

8

//Growth rates: negative growth implies population expansion

0

0

0

0

//Number of migration matrices : 0 implies no migration between demes

2

//Migration matrix 1

0 0 0 0

0 0 $MigA$ 0

0 0 0 0

0 0 0 0

//Migration matrix 0

0 0 0 0

0 0 0 0

0 0 0 0

0 0 0 0

//historical event: time, source, sink, migrants, new deme size, growth rate, migr mat index

6 historical event

$TIME3$ 3 2 1 $RESIZE4$ 0 0

$TENDBOT$ 2 2 0 $RESIZE3$ 0 0

$TBOT$ 2 2 1 $RESIZE2$ 0 0

$TIME2$ 1 2 1 1 0 1

$TIME1$ 0 1 1 $RESIZE1$ 0 0

$TDIV$ 0 1 1 $RESIZE$ 0 0

//Number of independent loci [chromosome]

1 0

//Per chromosome: Number of contiguous linkage Block: a block is a set of contiguous loci

1

//per Block:data type, number of loci, per gen recomb and mut rates

SNP 4410 0 1e-8

============================================================

============================================================

Scenario4.est

// Search ranges and rules file

// ****************************

//$NPOP0$

//$NPOP1$

//$NPOP2$

//$NPOP3$

[PARAMETERS]

//#isInt? #name #dist.#min #max

//all Ns are in number of haploid individuals

1 $NANC$ unif 40000 100000 output

1 $TDIV$ unif 300000 400000 output

1 $TIME1$ unif 9000 11000 output

1 $TIME2$ unif 700 1000 output

1 $TBOT$ unif 500 600 output

1 $TENDBOT$ unif 400 500 output

1 $TIME3$ unif 200 400 output

1 $N1_ANC$ unif 40000 100000 output

1 $N2_ANC$ unif 40000 100000 output

1 $NENDBOT$ unif 40000 100000 output

1 $NBOT$ unif 40000 100000 output

1 $NPOP0$ unif 40000 100000 output

1 $NPOP1$ unif 40000 100000 output

1 $NPOP2$ unif 40000 100000 output

1 $NPOP3$ unif 40000 100000 output

0 $MigA$ logunif 1e-10 1e-3 output

[RULES]

1 $TDIV$ > $TIME1$

1 $TIME1$ > $TIME2$

1 $TIME2$ > $TBOT$

1 $TENDBOT$ > $TIME3$

1 $NENDBOT$ < $NPOP2$

1 $NENDBOT$ < $NPOP3$

[COMPLEX PARAMETERS]

0 $RESIZE$ = $NANC$/$N1_ANC$ hide

0 $RESIZE1$ = $N1_ANC$/$NPOP0$ hide

0 $RESIZE2$ = $N2_ANC$/$NBOT$ hide

0 $RESIZE3$ = $NBOT$/$NENDBOT$ hide

0 $RESIZE4$ = $NENDBOT$/$NPOP2$ hide

Scenario4.tpl

//Parameters for the coalescence simulation program : simcoal.exe

4 samples to simulate :

//Population effective sizes (number of genes)

$NPOP0$

$NPOP1$

$NPOP2$

$NPOP3$

//Samples sizes and samples age

5

8

8

8

//Growth rates: negative growth implies population expansion

0

0

0

0

//Number of migration matrices : 0 implies no migration between demes

2

//Migration matrix 1

0 0 0 0

0 0 $MigA$ 0

0 0 0 0

0 0 0 0

//Migration matrix 0

0 0 0 0

0 0 0 0

0 0 0 0

0 0 0 0

//historical event: time, source, sink, migrants, new deme size, growth rate, migr mat index

6 historical event

$TIME3$ 3 2 1 $RESIZE4$ 0 0

$TENDBOT$ 2 2 0 $RESIZE3$ 0 0

$TBOT$ 2 2 1 $RESIZE2$ 0 0

$TIME2$ 1 2 1 1 0 1

$TIME1$ 0 1 1 $RESIZE1$ 0 0

$TDIV$ 0 1 1 $RESIZE$ 0 0

//Number of independent loci [chromosome]

1 0

//Per chromosome: Number of contiguous linkage Block: a block is a set of contiguous loci

1

//per Block:data type, number of loci, per gen recomb and mut rates

SNP 4410 0 1e-8

============================================================
